# Exome sequencing of 457 autism families recruited online provides evidence for autism risk genes

**DOI:** 10.1038/s41525-019-0093-8

**Published:** 2019-08-23

**Authors:** Pamela Feliciano, Xueya Zhou, Irina Astrovskaya, Tychele N. Turner, Tianyun Wang, Leo Brueggeman, Rebecca Barnard, Alexander Hsieh, LeeAnne Green Snyder, Donna M. Muzny, Aniko Sabo, The SPARK Consortium, Leonard Abbeduto, John Acampado, Andrea J. Ace, Charles Albright, Michael Alessandri, David G. Amaral, Alpha Amatya, Robert D. Annett, Ivette Arriaga, Ethan Bahl, Adithya Balasubramanian, Nicole Bardett, Asif Bashar, Arthur Beaudet, Landon Beeson, Raphael A. Bernier, Elizabeth Berry-Kravis, Stephanie Booker, Stephanie J. Brewster, Elizabeth Brooks, Martin E. Butler, Eric M. Butter, Kristen Callahan, Alexies Camba, Sarah Carpenter, Nicholas Carriero, Lindsey A. Cartner, Ahmad S. Chatha, Wubin Chin, Renee D. Clark, Cheryl Cohen, Eric Courchesne, Joseph F. Cubells, Mary Hannah Currin, Amy M. Daniels, Lindsey DeMarco, Megan Y. Dennis, Gabriel S. Dichter, Yan Ding, Huyen Dinh, Ryan Doan, HarshaVardhan Doddapaneni, Sara Eldred, Christine Eng, Craig A. Erickson, Amy Esler, Ali Fatemi, Gregory J. Fischer, Ian Fisk, Eric J. Fombonne, Emily A. Fox, Sunday Francis, Sandra L. Friedman, Swami Ganesan, Michael Garrett, Vahid Gazestani, Madeleine R. Geisheker, Jennifer A. Gerdts, Daniel H. Geschwind, Robin P. Goin-Kochel, Anthony J. Griswold, Luke P. Grosvenor, Angela J. Gruber, Amanda C. Gulsrud, Jaclyn Gunderson, Anibal Gutierrez, Melissa N. Hale, Monica Haley, Jacob B. Hall, Kira E. Hamer, Bing Han, Nathan Hanna, Christina Harkins, Nina Harris, Brenda Hauf, Caitlin Hayes, Susan L. Hepburn, Lynette M. Herbert, Michelle Heyman, Brittani A. Phillips, Susannah Horner, Jianhong Hu, Lark Y. Huang-Storms, Hanna Hutter, Dalia Istephanous, Suma Jacob, William Jensen, Mark Jones, Michelle Jordy, A. Pablo Juarez, Stephen Kanne, Hannah E. Kaplan, Matt Kent, Alex Kitaygorodsky, Tanner Koomar, Viktoriya Korchina, Anthony D. Krentz, Hoa Lam Schneider, Elena Lamarche, Rebecca J. Landa, Alex E. Lash, J. Kiely Law, Noah Lawson, Kevin Layman, Holly Lechniak, Sandra Lee, Soo J. Lee, Daniel Lee Coury, Christa Lese Martin, Deana Li, Hai Li, Natasha Lillie, Xiuping Liu, Catherine Lord, Malcolm D. Mallardi, Patricia Manning, Julie Manoharan, Richard Marini, Gabriela Marzano, Andrew Mason, Emily T. Matthews, James T. McCracken, Alexander P. McKenzie, Zeineen Momin, Michael J. Morrier, Shwetha Murali, Vincent J. Myers, Jason Neely, Caitlin Nessner, Amy Nicholson, Kaela O’Brien, Eirene O’Connor, Cesar Ochoa-Lubinoff, Jessica Orobio, Opal Y. Ousley, Lillian D. Pacheco, Juhi Pandey, Anna Marie Paolicelli, Katherine G. Pawlowski, Karen L. Pierce, Joseph Piven, Samantha Plate, Marc Popp, Tiziano Pramparo, Lisa M. Prock, Hongjian Qi, Shanping Qiu, Angela L. Rachubinski, Kshitij Rajbhandari, Rishiraj Rana, Rick Remington, Catherine E. Rice, Chris Rigby, Beverly E. Robertson, Katherine Roeder, Cordelia R. Rosenberg, Nicole Russo-Ponsaran, Elizabeth Ruzzo, Mustafa Sahin, Andrei Salomatov, Sophia Sandhu, Susan Santangelo, Dustin E. Sarver, Jessica Scherr, Robert T. Schultz, Kathryn A. Schweers, Swapnil Shah, Tamim Shaikh, Amanda D. Shocklee, Laura Simon, Andrea R. Simon, Vini Singh, Steve Skinner, Kaitlin Smith, Christopher J. Smith, Latha V. Soorya, Aubrie Soucy, Alexandra N. Stephens, Colleen M. Stock, James S. Sutcliffe, Amy Swanson, Maira Tafolla, Nicole Takahashi, Taylor Thomas, Carrie Thomas, Samantha Thompson, Jennifer Tjernagel, Bonnie Van Metre, Jeremy Veenstra-Vanderweele, Brianna M. Vernoia, Jermel Wallace, Corrie H. Walston, Jiayao Wang, Zachary Warren, Lucy Wasserburg, Loran Casey White, Sabrina White, Ericka L. Wodka, Simon Xu, Wha S. Yang, Meredith Yinger, Timothy Yu, Lan Zang, Hana Zaydens, Haicang Zhang, Haoquan Zhao, Richard A. Gibbs, Evan E. Eichler, Brian J. O’Roak, Jacob J. Michaelson, Natalia Volfovsky, Yufeng Shen, Wendy K. Chung

**Affiliations:** 1 Simons Foundation, New York, NY 10010 USA; 2 Department of Systems Biology, Columbia University, New York, NY 10032 USA; 3 Department of Genome Sciences, University of Washington School of Medicine, Seattle, WA 98195 USA; 4 Department of Psychiatry, University of Iowa Carver College of Medicine, Iowa City, IA 52242 USA; 5 Department of Molecular and Medical Genetics, Oregon Health & Science University, Portland, OR 97239 USA; 6 Human Genome Sequencing Center, Baylor College of Medicine, Houston, TX 77030 USA; 7 Howard Hughes Medical Institute, University of Washington, Seattle, WA 98195 USA; 8 Department of Pediatrics, Columbia University Medical Center, New York, NY 10032 USA; 9 MIND Institute and Department of Psychiatry and Behavioral Sciences, University of California, Davis, Sacramento, CA 95817 USA; 10 Division of Pediatric Psychology and Neuropsychology, Nationwide Children’s Hospital, Columbus, Ohio 43205 USA; 11 Department of Psychology, University of Miami, Coral Gables, FL 33146 USA; 12 Department of Pediatrics, University of Mississippi Medical Center, Jackson, MS 39216 USA; 13 Department of Psychiatry and Biobehavioral Sciences, University of California, Los Angeles, CA 90095 USA; 14 Department of Molecular and Human Genetics, Baylor College of Medicine, Houston, TX 77030 USA; 15 Vanderbilt Kennedy Center, Vanderbilt University Medical Center, Nashville, TN 37232 USA; 16 Department of Psychiatry and Behavioral Sciences, Rush University Medical Center, Chicago, IL 60612 USA; 17 Department of Psychiatry and Behavioral Sciences, University of Washington, Seattle, WA 98195 USA; 18 Department of Neurological Sciences, Rush University Medical Center, Chicago, IL 60612 USA; 19 Department of Pediatrics, Rush University Medical Center, Chicago, IL 60612 USA; 20 Department of Biochemistry, Rush University Medical Center, Chicago, IL 60612 USA; 21 Department of Psychiatry and Behavioral Neuroscience, Cincinnati Children’s Hospital Medical Center - Research Foundation, Cincinnati, OH 45229 USA; 22 Translational Neuroscience Center, Boston Children’s Hospital, Boston, MA 02115 USA; 23 Department of Psychiatry, University of North Carolina at Chapel Hill, Chapel Hill, NC 27599 USA; 24 Hugo W. Moser Research Institute at Kennedy Krieger, Baltimore, MD 21211 USA; 25 Department of Neurosciences, University of California, San Diego, School of Medicine, La Jolla, CA 92037 USA; 26 Department of Psychiatry and Behavioral Sciences, Emory Autism Center, Emory University, Atlanta, GA 30033 USA; 27 Center for Autism Research, Children’s Hospital of Philadelphia, Philadelphia, PA 19146 USA; 28 Genome Center, MIND Institute, Department of Biochemistry and Molecular Medicine, University of California, Davis, Sacramento, CA 95616 USA; 29 Department of Medicine, Boston Childrens Hospital, Boston, MA 02115 USA; 30 Department of Pediatrics, University of Minnesota, Minneapolis, MN 55414 USA; 31 Center for Autism and Related Disorders, Kennedy Krieger Institute, Baltimore, MD 21211 USA; 32 PreventionGenetics, Marshfield, WI 54449 USA; 33 Departments of Pediatrics & Psychiatry, Oregon Health & Science University, Portland, OR 97239 USA; 34 Department of Psychiatry, University of Minnesota, Minneapolis, MN 55455 USA; 35 Department of Pediatrics, University of Colorado School of Medicine, Aurora, CO 80045 USA; 36 Department of Pharmacology and Toxicology, University of Mississippi Medical Center, Jackson, MS 39216 USA; 37 Department of Pediatrics, University of California, San Diego, La Jolla, CA 92161 USA; 38 Department of Neurology, University of California, Los Angeles, CA 90095 USA; 39 Department of Pediatrics, Baylor College of Medicine, Houston, TX 77030 USA; 40 John P. Hussman Institute for Human Genomics, University of Miami Miller School of Medicine, Miami, FL 33136 USA; 41 Human Development & Family Studies, Colorado State University, Fort Collins, CO 80524 USA; 42 Department of Psychiatry, Weill Cornell Medicine, White Plains, New York, 10605 USA; 43 Department of Pediatrics, Vanderbilt University Medical Center, Nashville, TN 37232 USA; 44 Department of Health Psychology, University of Missouri, Columbia, MO 65211 USA; 45 Thompson Center for Autism and Neurodevelopmental Disorders, University of Missouri, Columbia, MO 65211 USA; 46 Geisinger Autism & Developmental Medicine Institute, Lewisburg, PA 17837 USA; 47 David Geffen School of Medicine, Semel Institute for Neuroscience and Human Behavior, University of California, Los Angeles, CA 90024 USA; 48 Research, Southwest Autism Research and Resource Center, Phoenix, AZ 85006 USA; 49 Department of Pediatrics, Boston Children’s Hospital, Boston, MA 02115 USA; 50 Department of Behavioral Sciences, Rush University Medical Center, Chicago, IL 60612 USA; 51 Department of Neurology, Boston Children’s Hospital, Boston, MA 02115 USA; 52 Maine Medical Center Research Institute, Portland, ME 04101 USA; 53 Greenwood Genetic Center, Greenwood, SC 29646 USA; 54 Department of Molecular Physiology & Biophysics, Vanderbilt University, Nashville, TN 37232 USA; 55 Department of Psychiatry, Columbia University Medical Center, New York, NY 10032 USA

**Keywords:** Behavioural genetics, Autism spectrum disorders, Medical genomics

## Abstract

Autism spectrum disorder (ASD) is a genetically heterogeneous condition, caused by a combination of rare de novo and inherited variants as well as common variants in at least several hundred genes. However, significantly larger sample sizes are needed to identify the complete set of genetic risk factors. We conducted a pilot study for SPARK (SPARKForAutism.org) of 457 families with ASD, all consented online. Whole exome sequencing (WES) and genotyping data were generated for each family using DNA from saliva. We identified variants in genes and loci that are clinically recognized causes or significant contributors to ASD in 10.4% of families without previous genetic findings. In addition, we identified variants that are possibly associated with ASD in an additional 3.4% of families. A meta-analysis using the TADA framework at a false discovery rate (FDR) of 0.1 provides statistical support for 26 ASD risk genes. While most of these genes are already known ASD risk genes, *BRSK2* has the strongest statistical support and reaches genome-wide significance as a risk gene for ASD (*p*-value = 2.3e−06). Future studies leveraging the thousands of individuals with ASD who have enrolled in SPARK are likely to further clarify the genetic risk factors associated with ASD as well as allow accelerate ASD research that incorporates genetic etiology.

## Introduction

Autism spectrum disorder (ASD) is an extremely variable condition characterized by deficits in social interactions and restrictive, repetitive behaviors. Currently, there are no FDA approved medications that address these core symptoms, despite the life-long morbidity and increased mortality in adults with ASD.^1^

Despite the significant clinical heterogeneity of this condition, many studies have shown that ASD is highly heritable, with genetic risk factors thought to explain the majority of the risk for ASD.^2^ Over the past decade, genomic studies focused on de novo, likely gene disrupting (dnLGD) variants (stopgain, frameshift, and essential splice site) have identified ~100 high-confidence ASD risk genes or loci.^3,4^ Previous studies have identified molecular diagnoses in 6–37% of individuals with ASD, with higher yields in individuals with additional co-morbidities that include intellectual disabilities, seizures, and other medical features.^5^

Here we describe the results of a pilot study that genetically characterized 457 families with one or more members affected with ASD enrolled online in SPARK.^6^ SPARK’s mission is to create the largest recontactable research cohort of at least 50,000 families affected with ASD in the United States for longitudinal phenotypic and genomic characterization who are available to participate in research studies. Using exome sequencing and genome-wide single nucleotide polymorphism (SNP) genotyping arrays, we identified variants that are the likely primary genetic cause of ASD in 14% of families. We also demonstrated that the genetic architecture in this self-reported cohort is similar to published, clinically confirmed ASD cohorts.^3,4,7^ Combining the SPARK data with prior studies, our analyses provide strong evidence that *BRSK2* is a high-confidence ASD risk gene (FDR *q*-value = 0.0015) and provide evidence that strengthens the association of additional genes (*FEZF2, ITSN1, PAX5, DMWD*, and *CPZ*) in ASD.

## Results

### Variant discovery

We report the exome sequencing and genotyping results of 1379 individuals in 457 families with at least one offspring affected with ASD, including 418 simplex and 39 multiplex families (Supplementary Fig. 1). Over 80% of participants are predicted to have European ancestry based on principal component analysis of common SNP genotypes (Supplementary Fig. 2). The male to female ratio is 4.4:1 among 418 offspring cases in simplex families, and 2.9:1 among 47 offspring cases in multiplex families. Of the 465 offspring affected with ASD, 25.6% also reported intellectual disability (Table 1). We identified 647 rare (allele frequency (AF) <0.001 in ExAC v0.3) de novo single nucleotide variants (SNVs) and indels (Supplementary Data 1) in coding regions and splice sites (1.4/offspring), including 85 likely gene disrupting (LGD) variants and 390 missense variants. Similar to the de novo variants identified from 4773 clinically ascertained ASD trios from previous studies,^3–8^ the frequency of dnLGD variants in the 465 affected offspring in SPARK (0.18/offspring) is 1.76-fold higher than the baseline expectation calculated by a previously published mutation rate model^9^ (*p*-value = 1.2 × 10^−6^ by one-sided exact Poisson test) (“Methods”; Supplementary Data 2).

**Table 1:** Phenotypic description of the 457 families with at least one offspring affected with ASD in the SPARK pilot study

All offspring with ASD								
Role	*n* = 465	Average age (years) of ASD diagnosis	Average age (years) at enrollment (SD)	Range of age (years) at enrollment	Intellectual disability (%)	Nonverbal (%)	Epilepsy (%)	ADHD (%)
Affected male offspring	376	4.8	12.9 (8.4)	1.5–44.6	22% (78/356)	13% (46/356)	7% (25/356)	30% (106/356)
Affected female offspring	89	5.6	12.8 (7.3)	1.9–29.8	33% (28/84)	10% (8/84)	13% (11/84)	23% (19/84)

1379 individuals in 39 multiplex and 418 simplex families were genomically characterized, including 472 individuals (465 offspring and 7 parents) affected with ASD. All phenotypic variables are not available for all participants

To identify de novo missense variants that are likely damaging, we applied two deleterious missense (D-mis) prediction algorithms on published ASD and SPARK de novo variants. Among the 390 de novo missense variants in affected offspring, 43.6% are predicted to be deleterious using CADD score ≥25^10^ and show 1.28-fold enrichment compared with baseline expectation in the general population (*p*-value = 6.6 × 10^−4^ by one-sided exact Poisson test). Using a more strict D-mis prediction algorithm with MPC score ≥2,^11^ 8% of de novo missense variants are predicted as deleterious and are enriched 1.88-fold in affected offspring which is comparable with the enrichment of dnLGD variants (*p*-value = 9.9 × 10^−4^ by one-sided exact Poisson test). The overall burden of de novo D-mis variants is similar to published studies (Supplementary Data 2).

Variants in constrained genes (pLI ≥ 0.5)^12^ explain most of the burden of dnLGD variants and de novo D-mis variants (defined by an MPC score ≥2) in the affected offspring in our study (Supplementary Data 2,b). Consistent with previous findings supporting the female protective model,^13^ we observed a nonsignificant trend toward a higher frequency of dnLGD variants in constrained genes in female cases compared with males (0.135/female vs 0.096/male), as well as higher frequency of de novo D-mis variants in female cases (CADD ≥25: 0.416/female vs 0.354/male, MPC ≥2: 0.09/female vs 0.066/male).

We also investigated deleterious inherited SNV/indel variants and found a modest excess of transmitted, rare LGD (AF < 0.001 in ExAC v0.3) variants observed only once among parents in our cohort (singletons) in constrained genes with pLI ≥ 0.5 (464 transmitted vs. 402 nontransmitted; rate ratio (RR) = 1.15, *p*-value = 0.038 by binomial test). Over-transmission of rare singleton LGD variants was not observed in genes that are not constrained (RR = 1.03, *p*-value = 0.31 by binomial test). The excess of transmitted singleton LGD variants in constrained genes increased after removing variants observed in the ExAC database (303 transmitted vs. 242 untransmitted; RR = 1.25, *p*-value = 0.010 by binomial test). These results provide further evidence that rare, inherited LGD variants in constrained genes confer increased risk for ASD.^14,15^ We then searched for known haploinsufficient ASD or neurodevelopmental disorder (NDD) genes (SFARI Gene score 1 or 2 or listed in DDG2P and associated with a neurological phenotype^16^) that are disrupted by the rare singleton LGD variants and are transmitted. We found 13 such variants (2 of them on the X chromosome), as compared with 10 variants that are not transmitted (including one on the X chromosome) (Supplementary Data 3). Manual review of these variants revealed that most of them are not likely pathogenic because they either affect only a subset of transcripts that are not expressed in the majority of tissues,^17^ are located close to the 3′ end of the transcript (last 5% of the coding sequence) or are indels that overlap but do not change the sequence of essential splice sites. The results suggest that the rare LGD variants in known ASD/NDD genes have only limited contribution to the overall transmission disequilibrium in this class of variants.

By integrating exome sequence read depth and SNP microarray signal intensity data, we identified 273 rare CNVs (occurring with a carrier frequency of ≤1% of the 1379 individuals in the analysis and also appear <1% in 1000 Genomes population and healthy controls^18^) in 206 affected offspring. Of these, 253 CNVs were inherited (0.544/affected offspring) and were on average 194 kb. These inherited CNVs contained an average of 3.7 genes, which reduces to an average of 0.7 genes that are constrained (pLI ≥ 0.5) (Supplementary Data 4). Similar to the frequency observed in previous studies^8,19^ (~5% within a cohort of affected individuals), we identified 20 de novo CNVs (dnCNVs) (0.043/affected offspring) (Supplementary Data 5). On average, dnCNVs were larger (1.6 Mb) and contained more total and constrained genes (19 genes, 5.5 constrained genes with a pLI ≥ 0.5).

Despite the fact we were underpowered to detect statistically significant burden differences between sexes, we observed a trend toward a 1.8-fold higher burden of dnCNVs in ASD females (0.067/female vs 0.037/male, respectively). In contrast, the frequency of rare, inherited CNVs in ASD females and males were similar (0.551/female vs 0.543/male, respectively). Similar to Sanders et al.^8^ dnCNVs in female cases also affect more genes than dnCNVs in males (2.3 vs 0.47 genes in dnCNVs per female proband vs per male proband, respectively; *p*-value = 0.013, Kruskal–Wallis test).

Of the CNVs detected, six mapped to the chromosome 16p11.2 region (three de novo and three inherited in five families). Four of the six 16p11.2 CNVs occurred at the most common breakpoints (BP4-BP5), occurring in 0.9% of affected offspring, consistent with the expected ASD prevalence.^20^ Together, the results suggest that the saliva-derived DNA collected in SPARK should provide comparable CNV data to previous studies using DNA derived from whole blood. We also used read-depth and SNP genotypes to identify several chromosomal aneuploidies (Supplementary Fig. 1), including one case of trisomy 21 (47, XY + 21), one case of Klinefelter syndrome (47, XXY), one case of Turner syndrome (45, X), and one case of uniparental iso-disomy of chromosome 6 (UPiD6).

Given their emerging role in genetic risk for ASD and other NDDs, we also assessed postzygotic mosaic mutations^21,22^ in the SPARK cohort. In parallel, we utilized a previously established method^23^ and a novel approach to identify likely mosaic SNVs (Methods, Supplementary Figs. 3–8). We identified 65 likely mosaic mutations (0.142/offspring) (Supplementary Data 6). The majority of these mutations were unique to the mosaic call set; however, 18 were also identified in the main de novo SNV call set with an average alternative allele fraction of 25.4% (Supplementary Data 6), suggesting that these mutations are likely to have occurred after fertilization. These results indicate that ~10% (65/652) of the total de novo SNVs in the SPARK pilot are of postzygotic origin. Comparing these data to a similar mosaic set from the Simons Simplex Collection (SSC),^23^ we found similar mosaic mutation characteristics, despite the fact that different DNA sources, capture reagents, and sequencing instruments were used (Supplementary Fig. 7). Due to the limited number of mosaic calls, we did not attempt to evaluate mosaic mutation burden. However, we observed that a number of potentially mosaic mutations were in known ASD/NDD genes or genes that are constrained (Supplementary Data 6). For example, we identified a potential mosaic LGD variant in *MACF1*, which is highly constrained (pLI = 1), plays essential roles in neurodevelopment, functions through the previously implicated Wnt signaling pathway,^24^ and has been recently suggested as a candidate gene based on a dnLGD variant in a Japanese ASD cohort.^25^ In *CREBBP*, which reached genome-wide significance in a recent NDD meta-analysis,^16^ we identified a potential mosaic missense variant, in addition to two other germline de novo missense variants in SPARK, adding to the evidence that it is an ASD/NDD risk factor. Future work will help determine the contribution of mosaic mutations in such genes to ASD.

### Genes with a higher mutational burden

We assessed genes with multiple dnLGD variants in the SPARK cohort and identified four genes with more than one dnLGD variant (*CHD8*, *FOXP1*, *SHANK3*, and *BRSK2*). *BRSK2* is the only gene with multiple dnLGD variants in SPARK that reached genome-wide significance (*p*-value = 2.3 × 10^−6^ by one-sided exact Poisson test, <0.05/20,000 genes), although there was one individual in the Autism Sequencing Consortium (ASC) cohort^7^ with a dnLGD variant in *BRSK2* (Table 2).

**Table 2:** Variants in four newly statistically significant and constrained (pLI ≥ 0.5) ASD risk genes (*BRSK2, ITSN1, FEZF2*, and *PAX5*) in published and SPARK trios and associated phenotypic information

**Subject ID**	**SP0037695**	**SP0042217**	**08C79336**	**SP0007556**	**SP0025011**	**SP0016887**	**13400.p1**	**13704.p1**	**14637.p1**	**SP0037344**	**11074.p1**	**SP0016232**	**12858.p1**
Cohort	SPARK	SPARK	ASC	SPARK	SPARK	SPARK	SSC	SSC	SSC	SPARK	SSC	SPARK	SSC
Gene	*BRSK2*	*BRSK2*	*BRSK2*	*ITSN1*	*ITSN1*	*ITSN1*	*ITSN1*	*ITSN1*	*ITSN1*	*FEZF2*	*FEZF2*	*PAX5*	*PAX5*
Variant	p.T547fs	c.951-1G > A	c.1365-1G > C	p.P1619L (MPC = 2.03)	p.P156fs	p.Q711X	c.1952 + 1del	p.E576*	p.P164Rfs*22	p.A397fs	p.R344C (MPC = 3.37)	p.E113V (MPC = 2.78)	p.A111fs
Inheritance	de novo	de novo	de novo	de novo	Inherited (paternal)	Inherited (paternal)	Inherited (maternal)	Inherited (paternal)	Inherited (paternal)	de novo	de novo	de novo	de novo
Confirmed by Sanger sequencing?	YES	YES	Not available	YES	YES	YES	Not available	Not available	Not available	YES	YES	YES	YES
Sex	Male	Male	Male	Male	Male	Female	Female	Male	Male	Male	Male	Male	Female
DSM diagnosis	ASD	ASD	ASD	Asperger’s disorder	Asperger’s disorder	ASD	Autism	PDD-NOS	Autism	ASD	Autism	ASD	Autism
Age at evaluation (years)	8	19	5.3	30	34	2	4.2	4.3	5.4	23	9	14	4
Medical concerns	None	Premature birth (24 weeks), vision/hearing problems (not specified)	Unknown	Obesity, vision/hearing problems (not specified)	None	None	Migraines	None	None	None	None	Neurological problems (not specified), sleep disorder	None
Seizures (TRUE/FALSE)	FALSE	TRUE	FALSE	FALSE	FALSE	FALSE	FALSE	FALSE	FALSE	FALSE	Febrile seizures only	FALSE	FALSE
Intellectual Disability (TRUE/FALSE), IQ (if known)	TRUE	TRUE, ≤25	Unknown	FALSE, ≥129	FALSE	FALSE	FALSE, FSIQ = 116	FALSE FSIQ = 91	TRUE− mild, FSIQ = 63	FALSE	TRUE, FSIQ = 68	TRUE, 55–69	FALSE, FSIQ = 91
Language level (at age of evaluation)	Delayed, single words	Delayed, no words	Delayed	Fluent speech (sentences)	Fluent speech (sentences)	Delayed, single words	Delayed, phrase speech (ADOS mod. 2)	Delayed, phrase speech (ADOS mod. 2)	Delayed, phrase speech (ADOS mod. 2)	Unknown	Fluent speech (sentences; ADOS mod. 3)	Delayed, no words	Delayed, phrase speech (ADOS mod. 2)
Language regression (TRUE/FALSE)	FALSE	FALSE	Unknown	FALSE	Unknown	TRUE	FALSE	TRUE	FALSE	Unknown	TRUE	TRUE	TRUE
Co-morbid psychological diagnoses	Learning disorder, motor skills delay, speech articulation problems, feeding disorder	Motor skills delay, speech articulation problems, feeding disorder, encopresis, enuresis	None	Attention or behavior problems—not specified, mood or anxiety problems—not specified, feeding disorder	Attention or behavior problems—not specified, OCD	Feeding disorder	Anxiety	None	None	None	None	Learning disorder, feeding disorder	None
Early motor delay (TRUE/FALSE)	FALSE	TRUE	FALSE	FALSE	Unknown	FALSE	FALSE	FALSE	FALSE	Unknown	FALSE	FALSE	FALSE

All damaging variants in SPARK participants within these genes have been confirmed with Sanger sequencing. Damaging variants in *PAX5* and *FEZF2* in the SSC were previously validated.^3^ MPC scores are listed for missense mutations. All phenotypic information for SPARK participants was collected online

**Fig. 1:**
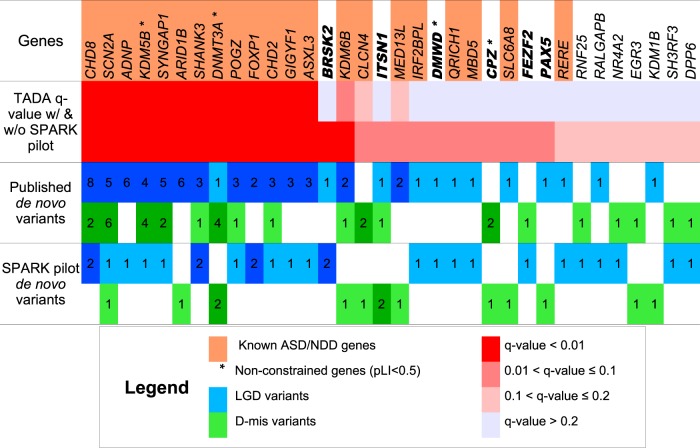
Meta-analysis using the TADA framework identifies 34 genes with a false discovery rate (FDR) of ≤0.2. Known ASD genes are defined as those with SFARI Gene^105^ score ≤2 or implicated in a previous TADA meta-analysis (FDR ≤ 0.1)^8^ and known NDD genes are those listed in the DDG2P database^16^ and are colored orange. Deleterious missense (D-mis) variants are defined by CADD score ≥25. A total of 34 genes with at least one de novo damaging variant observed in SPARK pilot trios achieve an FDR ≤ 0.2 after meta-analysis with published trios (total *n* = 5238). Fourteen genes are not classified as known ASD or NDD genes. Six genes (*BRSK2, ITSN1, FEZF2, PAX5, DMWD*, and *CPZ*) that have an FDR ≤ 0.1 only after inclusion of SPARK de novo variants are highlighted. The asterisk symbol indicates genes that are not constrained (pLI < 0.5)

To increase the statistical power to identify new ASD genes, we performed a meta-analysis of de novo variants in 4773 published ASD trios^3,4,7,8^ and 465 SPARK trios using TADA^26^ (Methods). In this analysis, we included dnLGD variants and de novo D-mis variants, which we defined as those that have a CADD score ≥25.^10^ The TADA analysis presumes a model of genetic architecture compatible with the observed burden and recurrence of de novo damaging variants and assigns a false discovery rate (FDR) *q*-value for each gene based on the number of damaging variants and baseline mutation rates. We identified 67 genes with an FDR threshold of ≤0.1. Of these, there are 26 genes that also harbored a damaging variant in SPARK, most of which are already known ASD/NDD genes. There are six genes (*BRSK2*, *ITSN1*, *PAX5, FEZF2, DMWD*, and *CPZ*) that reached an FDR threshold of 0.1 only after the inclusion of de novo variants from SPARK (Fig. 1). The association signal for *DMWD* was driven by two LGD variants but the gene is not constrained (pLI = 0), so this gene may be a false positive.

Of the 34 genes listed in Fig. 1, only *BRSK2* meets genome-wide significance as a new ASD risk gene. All four individuals in SPARK, ASC and the SSC with de novo functional variants in *BRSK2* are males with cognitive impairment and severe speech delay (Table 2). *MBD5* and *IRF2BPL* reached an FDR value of ≤0.1 in a previous meta-analysis but not significant in our analysis of published de novo variants,^8^ because the previous study also included evidence from de novo CNVs and deleterious variants of unknown inheritance from a case-control sample in that analysis. *MBD5*, as well as *QRICH1, SLC6A8*, and *RERE* are known NDD risk genes in the latest DDG2P database.^16^

In our TADA results, we further broadened our focus on genes that harbored damaging variants in the SPARK data and those that had a FDR ≤ 0.2 (Supplementary Data 7). When the TADA analysis is restricted to genes harboring damaging variants in SPARK with an FDR ≤ 0.2, we identified 34 genes (Fig. 1), of which 21 have a known role in ASD or NDDs. We also incorporated inherited variants and CNVs from the SPARK families into the TADA analysis, but did not find additional newly significant genes.

We then searched for additional supporting evidence for a role of these genes in ASD and NDDs, including other deleterious variants in previous studies and case reports not included in the meta-analysis, membership in gene sets previously associated with ASD,^3,4,7,8^ and published functional studies (Supplementary Data 8). Recent studies have reported additional individuals with ASD and/or NDD with de novo damaging variants in these genes including *BRSK2*,^27^ *PAX5*,^4,28^ *NR4A2*,^29,30^ *RALGAPB*,^7,31,32^ and *DPP6*.^5,33,34^

In addition to multiple deleterious variants in these candidate ASD risk genes, we also found evidence that they function in biological pathways previously linked to ASD. For example, mRNA translation of *BRSK2, ITSN1*, and *RALGAPB* in neurons is predicted to be regulated by FMR1 protein.^35^ In addition, *ITSN1* and *DPP6* are part of the postsynaptic density components in human neocortex.^36^ *PAX5* and *FEZF2* are involved in transcription regulation during central nervous system development.^4,24,37^ *KDM1B* is a known chromatin modifier, and *EGR3* has been implicated in neurodevelopment.^38,39^

We also searched rare singleton inherited LGD variants of these newly significant genes in SPARK and published SSC data, and identified five additional cases (three in SSC, two in SPARK) carrying inherited LGD variants of *ITSN1* that likely cause loss of gene function. Interestingly, of the six ASD cases with LGD variants in *ITSN1*, five do not have intellectual disability (Table 2). The less severe phenotype and inheritance from unaffected parents are consistent with the modest effect size, although future studies will help determine if *ITSN1* is a bona fide ASD risk gene. Furthermore, in ASC case-control samples,^7^ LGD variants in *ITSN1* were also identified in the controls (three in 5397 ASC controls and comparable with the cumulative AF of 2.5e−4 in gnomAD v2.1), although they were still overrepresented in cases (two in 1601 cases).

### Functional network analysis and gene expression patterns in candidate ASD risk genes

To relate the candidate ASD risk genes identified in our TADA analysis to previous knowledge of integrated gene networks in ASD, we scored genes with a TADA FDR ≤ 0.2 and not currently listed in SFARI Gene using forecASD, a new ensemble classifier that integrates spatiotemporal gene expression, heterogeneous network data, and previous gene-level predictors of ASD association.^40^ Using forecASD, we derived a single score that ranks the evidence for each gene to be involved in ASD risk. Using this approach, we identified ten genes (*RNF25, DMWD, CLCN4, ITSN1, CPZ, SH3RF3, EGR3, RALGAPB, KDM1B*, and *BRSK2*) that have a TADA FDR ≤ 0.2 and were not listed in the SFARI Gene database. These genes have significantly elevated forecASD scores (*p*-value = 0.007, *Z*-test in logistic regression model controlling for contribution of previous TADA scores; Supplementary Fig. 9). Furthermore, two predictive features in forecASD that summarize brain expression support and network support are also found to be significantly elevated over the genome background in the set of these ten genes (*p*-value = 0.015 and *p*-value = 0.03, respectively, Wilcoxon test; see Supplementary Fig. 9). Importantly, neither of these metrics uses genetic data directly, so these genes collectively have support across the three independent and distinct domains of genetic, network, and brain expression evidence. These statistical associations are conservative estimates because they compare the distribution of evidence scores among the candidate genes described here to the remainder of the genome, which includes well-established ASD genes. Eight of these genes, *BRSK2, KDM1B, RALGAPB, EGR3, SH3RF3, CPZ, ITSN1*, and *CLCN4* fall in the top decile of forecASD scores (the top decile being a recommended cutoff used to define probable ASD risk genes), supporting these genes as having similar properties overall compared with known ASD risk genes.

**Fig. 2:**
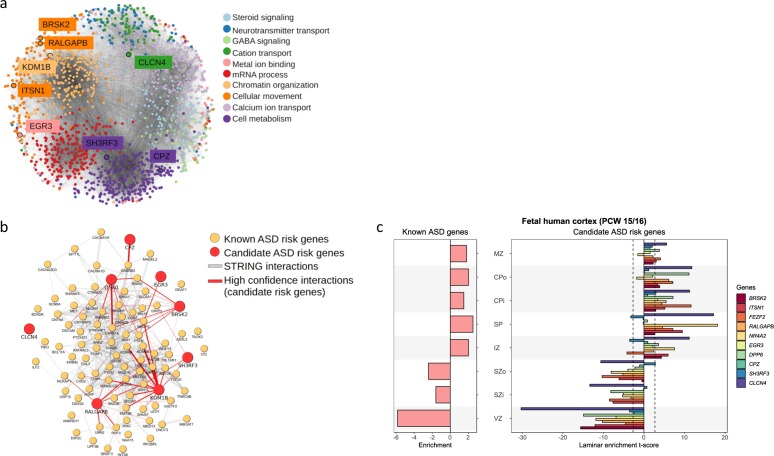
Network analysis and gene expression of candidate ASD risk genes. **a** STRING networks of forecASD genes, **b** STRING networks of known ASD genes, and **c** gene expression of human fetal cortex at postconceptual weeks (PCW) 15–16. Known ASD genes are defined as those with a SFARI Gene scores^104^ ≤2 (84 genes, indicated as SFARI) or implicated in a previous TADA meta-analysis^8^ at an FDR ≤ 0.1 (65 genes, indicated as TADA). The enrichment for each gene was measured by the *t*-statistics comparing the expression level in each layer against all other layers. The enrichment of a gene set is the mean of *t*-statistics of its genes. Two candidate ASD risk genes (*PAX5* and *KDM1B*) are not shown due to the low expression levels in human developing cortex (RPKM <1 for at least 20% available neocortical samples in BrainSpan^48^). Data were extracted from Supplementary Tables of Parikshak et al.^45^ Laminae abbreviations: marginal zone (MZ), outer/inner cortical plate (CPo/CPi), subplate (SP), intermediate zone (IZ), outer/inner subventricular zone (SZo/SZi), ventricular zone (VZ)

To illustrate the network context of these eight candidate ASD risk genes, we clustered them along with genes scoring within the top decile of forecASD (Fig. 2a). Network analysis yielded ten tightly connected clusters with distinct biological functions (Supplementary Data 9). Several genes were assigned to clusters that showed enrichment for gene sets consistent with their known functions, including *KDM1B*,^41^ *BRSK2*,^42^ and *ITSN1*^43^ consistent with published functional evidence (Supplementary Data 8). In a subsequent analysis, the interactions between known and novel ASD candidate risk genes were visualized (Fig. 2b). This subnetwork was significantly interconnected (*p*-value = 5.0 × 10^−179^ by hypergeometric test), with novel genes showing significantly more functional associations with known ASD candidate risk genes than expected by chance (*p*-value = 0.005 by hypergeometric test).

Using coexpression networks seeded by high-confidence ASD risk genes, a previous study found that cortical projection neurons in layers V and VI of the human midfetal prefrontal and primary motor-somatosensory cortex are a key point of convergence for ASD risk genes.^44^ Another study also showed that unbiased gene co-expression networks overrepresented with candidate ASD risk genes are more highly expressed in the cortical plate and subplate laminae of the developing human cortex, which will go on to form mature layers II–VI of the cerebral cortex.^45^ One of the newly statistically significant genes we identified, *FEZF2*, is a powerful master regulator gene critical for establishing corticospinal neurons,^46^ which connect layer Vb of the cortex to the spinal cord, and is known to be expressed in the putative layer V in the late mid-fetal human cortex.^47^

We evaluated gene expression of the candidate ASD risk genes identified by either the TADA meta-analysis and forecASD with regard to cortical layer specificity in the human developing brain.^48^ Ten of these genes (*BRSK2, ITSN1, FEZF2, RALGAPB, NR4A2, EGR3*, *DPP6, CPZ, SH3RF3*, and *CLCN4*) have expression data in developing fetal human cortex, and similar to Parikshak et al.^45^ they show a trend of increased expression at postconceptual week (PCW) 15–16 (Fig. 2c) and PCW 21 (Supplementary Fig. 10) in the cortical plate and subplate laminae, which will form layers II–VI of the mature cerebral cortex. The mean of *t*-statistics of these ten genes in the inner cortical plate (CPi) and subplate (SP) are greater than two standard deviations (SD) from the mean of randomly selected genes matched for gene length and GC content (*P* < 0.01 by simulation).

We further evaluated cell-type specificity using recently published single-cell RNA-seq data from fetal and adult mouse and human brains^49^ (Supplementary Figs. 11 and 12), and found the expression specificity of these candidate ASD risk genes is highest in pyramidal neurons in the mouse hippocampus CA1 region with an enrichment of 3.4 SD from the bootstrapped mean (*p*-value = 9.6e−3 by simulations controlling gene length and GC content, Supplementary Fig. 11). The enrichment in pyramidal neurons is also observed in the hippocampus CA1 region in human (2.4 SD above the bootstrapped mean, *p*-value = 0.02 by simulation) using recently published human single nucleus RNA-seq data.^50^ These results are consistent with a previous study showing that ASD protein–protein interaction networks related to the 16p11.2 CNV display significantly enriched expression during mid-fetal development as well as early childhood in cerebral cortex.^51^ Taken together, we find that the candidate ASD risk genes identified in this study demonstrate differential expression patterns similar to that of known ASD risk genes, providing further support that these genes function in similar biological pathways and mechanisms as known ASD risk genes.

### Diagnostic yield in SPARK

Families in the pilot study were selected without regard to genetic diagnosis. Thirteen of the 457 families self-reported a genetic diagnosis, and all were confirmed by our analyses and serve as positive controls to validate our genomic analyses (Supplementary Data 10). For the remaining 444 families, we identified 50 (10.4%) deleterious genetic variants (8 dnCNVs, 14 inherited CNVs, 23 de novo SNVs or indels, 3 inherited LGD variants and 2 chromosomal aneuploidies) in known ASD risk genes or loci in 49 affected individuals (Supplementary Data 10). We also identified an additional 19 likely deleterious genetic variants (1 dnCNV, 1 inherited CNV, 14 de novo SNVs and 3 inherited SNVs) in possible ASD risk genes or loci in an additional 14 individuals (3.4%). For all cases, we defined deleterious ASD-associated variants as those meeting likely pathogenic or pathogenic criteria according to ACMG standards.^52^ We defined possible ASD-associated variants as either SNVs that are de novo missense variants that affect known NDD or ASD genes and have an MPC score^11^ ≥2, loss-of-function variants that disrupt possible NDD or ASD genes, or CNVs that delete one or more possible NDD or ASD genes or duplicate known ASD or NDD loci. Possible ASD genes include those that are newly significant in this study (FDR ≤ 0.2) with independent evidence from literature, or genes implicated by other studies with multiple LGD variants found in affected ASD and NDD cases (summarized Supplementary Data 8). We did not search for or discover any incidental findings unrelated to ASD in these families. When DNA was available, ASD-associated genetic findings were confirmed by Sanger sequencing or chromosome microarray, and genetic results were returned to the families (*n* = 28).

## Discussion

Overall, the genomic characterization of 457 ASD families (418 simplex and 39 multiplex) in SPARK implicates a number of candidate risk genes in ASD that converge on similar biological networks as known ASD risk genes. We identified a returnable genetic result related to ASD in 10.4% of affected offspring and have begun returning individual genetic results to the families after confirming results in a clinical laboratory. Not surprisingly, our diagnostic yield was highest in affected individuals who also report presence of seizures (27%). The yield in individuals who also report intellectual disability was also higher (20%) than the overall cohort.

In our analysis, our diagnostic yield in affected offspring in multiplex families (15.2%) was slightly higher than affected offspring in simplex families (10.1%). Interestingly, the genetic findings in multiplex families rarely explained ASD in all affected family members (Supplementary Fig. 13), similar to previous studies that have also found affected siblings with discordant mutations.^53,54^ For example, in a family with an affected father and three affected children, the most severely affected child harbored a dnLGD in *ADNP*. No other family member carried this variant or any other identifiable contributing variant. In another pedigree, an affected male child with an affected father inherited a 15q11.2 BP1-BP2 deletion from a mother who does not report an ASD diagnosis, but we found no contributing variant in the affected father. We also identified eight families in which there was greater than one contributing variant, even in families in which we were unable to identify contributing variants in all affected offspring. In one family with two affected children, the female child inherited a 1q21.1 CNV from an unaffected mother and also harbored a dnLGD in *RALGAPB*. However, the affected male child did not harbor either of these variants and the CNV identified, like many potentially pathogenic variants, is known to be variably expressive. Future studies with larger sample sizes will allow for a more robust comparison of the genetic architecture of ASD in simplex vs. multiplex families.

Over time, we expect the diagnostic yield in SPARK to increase as more individuals with ASD are studied and as additional genetic risk factors are identified. For example, we identified LGD variants in *MEIS2* and *AKAP10* and deletions of the *NFIB, DLL1*, and *HNRNPD* genes. Although these genes did not reach statistical significance in our TADA meta-analysis, their role in ASD is supported by multiple mutations in the literature, and they likely represent other candidate ASD risk genes (Supplementary Data 8). We interpreted those variants as possible contributors to ASD in those individuals. The genetic findings in those cases will be confirmed and returned in the future if and when these genes are established as ASD risk genes.

Using a systems biology approach, we demonstrated that the newly statistically significant and candidate ASD risk genes identified in this analysis are well-supported beyond genetic association and are predicted to be ASD risk genes based on a variety of functional properties, including patterns of spatiotemporal gene expression in the brain and protein network connectivity. *BRSK2* and seven of the candidate ASD risk genes scored in the top decile of forecASD, an integrator of published functional evidence for ASD risk genes (Supplementary Fig. 9). The genes localized to network clusters representing processes critical for neurodevelopment (Fig. 2), including chromatin modification (*KDM1B*), neuronal polarity^55^ (*BRSK2*), and neuronal migration of pyramidal neurons^56^ (*ITSN1*). The candidate ASD risk genes also showed significant over-connectivity to known ASD risk genes (*p*-value = 0.005 by hypergeometric test, Fig. 2b). Together, the TADA genetic association analysis coupled with the supporting functional and network-level data triangulate these genes as being robust and biologically plausible contributors to ASD risk.

Despite the limited sample size in this pilot study, we were able to identify four newly statistically significant ASD genes. Power analysis using a simulation-based approach confirmed that the observed yield is expected given the presumed genetic architecture in the TADA analysis (Supplementary Table 1). We expect to identify ~70–75% of all ASD risk genes in the future that meet a similar FDR threshold (0.1–0.2) when we reach SPARK’s goal of sequencing 50,000 complete trios (Supplementary Table 1). Other analyses of large cohorts in ASD are underway, including a recent analysis of ~12,000 individuals with ASD.^57^ This study, which used a mixture of family-based and case-control data, found statistical support for 99 ASD risk genes, increasing the number of ASD risk genes from 65.^8^ Future meta-analyses of both SPARK data and other ASD cohort data are planned to maximize ASD risk gene discovery.

For many genes identified with de novo damaging variants, inherited loss-of-function variants in affected individuals were not found (Kosmicki et al. ^15^ and this study), suggesting our current knowledge about ASD risk genes is biased toward those with high penetrance. Future studies with larger sample sizes will be needed to identify and validate additional risk genes of lower penetrance that confer inherited ASD risk.

Altogether, these data suggest that the methods used to ascertain individuals with ASD, saliva collection, and genomic data are of high quality, and future analysis of the tens of thousands of families enrolling in SPARK will significantly contribute to our understanding of the genetic basis of ASD. By returning genetic results to participants, we expect to increase engagement and increase the number of recontactable participants for genetically targeted clinical research and trials.

## Methods

### Participant recruitment, phenotyping, and DNA sequencing

All participants were recruited to SPARK under a centralized IRB protocol (Western IRB Protocol #20151664). All participants provided written informed consent to take part in the study. Written informed consent was obtained from all legal guardians or parents for all participants age 18 and younger and all participants age 18 and older who have a legal guardian. Assent was also obtained from dependent participants age 10 and older. Participants are asked to fill out questionnaires online as described here: https://www.sfari.org/spark-phenotypic-measures/. Families are classified as multiplex if the initial individual with ASD registered in the study has a first-degree family member with ASD, as indicated either by enrollment or survey report.

Essential phenotypic information was curated across language and motor development, co-morbidities, and Repetitive Behavior Scale-Revised,^58^ Social Communication Questionnaire-Lifetime^59^ and Developmental Coordination Disorder Questionnaire score^60^ (Table 2). In SSC, all phenotype details were determined through clinic evaluation and interview; specifically, language delay was defined by Autism Diagnostic Observation Schedule module (1–4) per age,^61^ and regression was determined from the Autism Diagnostic Interview-Revised.^62^ For SPARK, all variables were taken from parent report. It was noted that rates of language disorder and psychiatric co-morbidities are lower in SSC likely due to DSM-IV diagnostic practice at the time.

Saliva was collected using the OGD-500 kit (DNA Genotek) and DNA was extracted in a CLIA-certified laboratory at the Baylor Miraca Genetics Laboratories (Houston, TX) or PreventionGenetics (Marshfield, WI). Exome capture was performed using VCRome and the spike-in probe set PKv2 at the Baylor College of Medicine Human Genome Sequencing Center (Houston, TX). Captured exome libraries were sequenced using the Illumina HiSeq platform in 100 bp paired end reads. Samples were sequenced to a minimum standard of >85% of target covered at 20×, and on average, 96% of the target was sequenced to 20×. The Illumina HumanCoreExome (550K SNP sites) array was used for genotyping.

### Read alignment and QC

Postsequencing reads were aligned to build 37 of the human genome using bwa version 0.6.2-r126,^63^ duplicates were marked using Picard version 1.93 MarkDuplicates, and indels were realigned using GATK^64^ version 2.5-2-gf57256b IndelRealigner. Quality checks were performed on the BAM files using SAMTools^65^ version 1.3.1 flagstat and Picard version 2.5.0 CalculateHsMetrics. Overall, 98 ± 1.8% of the reads mapped to the genome, 96 ± 2.3% of the reads were properly paired reads, and 87 ± 15% of targeted regions had ≥10× coverage.

KING^66^ was used for relatedness inference based on the genotype of exome SNPs (MAF >0.01). Estimated kinship coefficient and number of SNPs with zero shared alleles (IBS0) between a pair of individuals were plotted. Parent–offspring, sibling pairs, and unrelated pairs can be distinguished as separate clusters on the scatterplot (Supplementary Fig 1). One outlier parent–offspring pair (SP0002452 and mother) showed higher than expected IBS0 and was caused by parental chr6 iso-UPD. Pairwise scatterplots of heterozygotes to homozygotes (het/hom) ratio of chromosome X, sequencing depth of chromosome X and Y normalized by the mean depth of autosomes were used for sex check. Two samples with sex chromosome aneuploidy were identified as outliers in the scatterplot (Supplementary Fig. 2).

### Variant calling

#### De novo SNV/indel detection

De novo sequence variants were called by three groups—University of Washington (UW), Simons Foundation (SF), Columbia University Medical Center (CUMC)—according to the methods below.

#### UW

Variants were called from whole exome sequence (WES) using FreeBayes^67^ and GATK.^64^ FreeBayes version v1.1.0-3-g961e5f3 was used with the following parameters: –use-best-n-alleles 4 -C 2 -m 20 -q 20; and GATK version 3.7 HaplotypeCaller was used with the following parameters: -A AlleleBalanceBySample -A DepthPerAlleleBySample -A MappingQualityZeroBySample -A StrandBiasBySample -A Coverage -A FisherStrand -A HaplotypeScore -A MappingQualityRankSumTest -A MappingQualityZero -A QualByDepth -A RMSMappingQuality -A ReadPosRankSumTest -A VariantType. Postcalling bcftools^68^ version 1.3.1 norm was used with the following parameters -c e -O z -s -m –both. We identified candidate de novo calls based on the intersection of FreeBayes and GATK VCF files and identifying variants present in offspring but not in parents. We required a minimum of ten sequence reads in all members of the parent–offspring trio; an allele balance >0.25 and a PHRED quality >20 for both FreeBayes and GATK variants.

#### SF

Sequence data were preprocessed using GATK best practices and variant calls were predicted using three variant callers: GATK v3.6,^69^ FreeBayes v1.1.0-441, and Platypus v0.8.1-0.^70^ GATK: gVCF files were generated for each sample with GATK HaplotypeCaller (minimum confidence thresholds for calling and emitting was set to 30 and 10, respectively); joint variant calls were performed using GATK GenotypeGVCFs with the recommended default hard filters. For SNPs, we filtered out: QD <2.0 || FS >60.0 || MQ <40.0 || MQRankSum <−12.5 || ReadPosRankSum <−8.0. For indels, we filtered out: QD <2.0 || FS >200.0 || ReadPosRankSum <−20.0. FreeBayes: variants were called with default settings for optimal genotyping of indels in lower-complexity sequence. The final data set included candidate calls with a quality of 5 or greater. Platypus: variant calling was performed with local assembly analysis when at most ten haplotypes were allowed. Variants were filtered out for allele bias (*p*-value < 0.0001), bad reads (>0.9), sequence complexity (>0.99) and RMSMQ (<20); other filters were applied on estimated haplotype population frequency (FR), total coverage at the locus (TC) and phred-scaled quality of reference allele (QUAL): (FR[0] < = 0.5 and TC < 4 and QUAL < 20),or (TC < 13 and QUAL < 10),or (FR[0] > 0.5 and TC < 4 and QUAL < 50). For each variant caller, a variant was identified as a candidate de novo variant if the variant was called in the proband and it occurred only once in the cohort, with an alternative allele fraction between 0.2 and 0.8. Both parents were required to have the homozygous reference genotype at the de novo locus. Read coverage of the variant locus had to be at least ten reads in each sample in the trio. De novo candidate variants were classified by DNMFilter algorithm^71^ that was retrained with the SSC data set^3,14^: 1800 de novo mutations identified by both Iossifov et al.^3^ and Krumm et al.,^14^ 1104 validated SNVs and indels from both studies and 400 variants that failed validation. We also randomly selected ~3000 negative examples from the pool of all SSC variants that were not confirmed to be de novo. After merging de novo candidate variants from three variant callers, candidate de novos were considered if they occurred only once in the cohort, passed hard filters, and had assigned de novo probability greater than 0.88 for SNVs and greater than 0.0045 for small indels. In the latter case, the total parental alternative allele count <3 reads.

#### CUMC

Variants were called from aligned sequence data using GATK HaplotyperCaller to generate individual level gVCF files. All samples in the cohort were then jointly genotyped and have variant quality recalibrated by GATK v3.8.^64^ A variant present in the offspring with homozygous reference genotypes in both parents was considered to be a potential de novo variant. We used a series of filters to identify de novo variants. Briefly, we included variants that passed VQSR filter (tranche ≤ 99.7 for SNVs and ≤99.0 for indels) and had GATK’s Fisher Strand ≤ 25, quality by depth ≥2. We required the candidate de novo variants in probands to have ≥5 reads supporting the alternative allele, ≥20% alternative allele fraction, Phred-scaled genotype likelihood ≥60 (GQ), and population AF ≤0.1% in ExAC; and required both parents to have ≥10 reference reads, <5% alternative allele fraction, and GQ ≥ 30.

#### De novo SNV/indel consensus call set and annotation

De novo variants were independently called by three centers—UW, SF, CUMC. De novo variants called by all three groups were included in the final list by default. Those called by one or two groups were manually evaluated and included in the final list if consensus was reached among all groups after discussion and manual inspection with IGV plots. Variants were annotated by ANNOVAR^72^ based on GENCODE Basic v19.^73^ Candidate variants in the ACMG secondary findings v2 59 gene list^74^ (except *PTEN*, *TSC1*, and *TSC2*) were excluded. Coding de novo variants—nonsense, missense, or synonymous SNVs, frameshift or nonframeshift indels, and splicing site variants—were annotated. De novo variants were also annotated with snpEff version 4.1g^75^ (reference GRCh37.75), SFARI Gene scores (version q1, 2018, https://gene.sfari.org/database/gene-scoring/), CADD,^10^ MPC^11^ and findings from Deciphering Developmental Disorders project (gene2phenotype).^16^

#### Inherited singleton variants

We first performed following filtering on individual genotypes. We required minimal read-depth ≥10 and GQ ≥30, required allelic balance <0.1 for homozygotes reference, >0.9 for homozygotes alternative, and 0.3–0.7 for heterozygotes SNVs (0.25–0.75 for heterozygotes indels). Genotype calls not passing those criteria were set to missing. Then we removed variants having missing genotypes in >25% of founders. We focused analysis on singleton variants in which the alternative allele was only seen in one parent in the data. We calibrated GATK’s VQS LOD score for SNV and indels separately such that synonymous singleton SNVs and nonframeshift singleton indels were transmitted 50% of the time (Supplementary Fig. 14) The resulting VQS LOD score cutoffs are −1.85 for SNVs and −1.51 for indels. As mentioned in the Results section, inherited LGD variants are less likely to cause a complete loss of function to the gene. To prioritize inherited LGD variants, we require the variant to be annotated as HC (high-confidence) by LOFTEE v0.3^12^ using default parameters in >60% of the GENCODE transcripts.

#### Identification of mosaic mutations

Mosaic SNVs were independently called by two centers—Oregon Health & Science University (OHSU) and CUMC. The OHSU approach was previously published^23^ and utilized a binomial deviation and logistic regression model to score candidate mosaic variants. The CUMC approach used a novel approach that was based on a beta-binomial deviation and an FDR based approach to determine per site thresholds.

#### OHSU

SNVs were called as previously described.^23^ In brief, pileups were generated using SAMtools (v 1.1) with BAQ disabled and mapQ 29 (*samtools mpileup –B –q 29 –d 1500*) on processed BAMs. Variants were called on individual samples using VarScan 2.3.2, LoFreq 2.1.1 and an in-house mpileup parsing script (mPUP). Additional parameters for Varscan included: –*min-var-freq 1* *×* *10*^*−15*^ *–p-value 0.1*. Per sample caller outputs were combined and annotated using ANNOVAR (03/22/15 release) with databases: Refseq genes (obtained 03/2017), segmental duplications (UCSC track genomicSuperDups, obtained 03/25/2015), repetitive regions (UCSC track simpleRepeat and hg19_rmsk, obtained 03/25/2015), Exome Aggregation Consortium (ExAC) release 0.3 (obtained 11/29/2015), Exome Sequencing Project (ESP) 6500 (obtained 12/22/2014), and 1000 Genomes Phase 3 version 5 (obtained 12-16-2014).

Variants were filtered based on the best practices established in Krupp et al.:^23^ (1) variant must be exonic or disrupt a canonical splice site, (2) have a population frequency of ≤0.5%, (3) have at least five alternative reads, (4) not be in a known segmental duplication or repetitive regions (SDTRF), (5) called by at least two variant callers, (6) SPARK cohort count ≤1 and SSC cohort count ≤2, (7) variant read mismatch ≤3, and (8) allele fraction upper 90% confidence interval ≤0.05. For a variant to be considered de novo, parental alternative allele count must be ≤4 reads. De novo variants were considered to be candidate mosaic variants if: (1) the probability the allele fraction significantly deviated from heterozygous (PHET) was ≤0.001, (2) the allele fraction upper 90% confidence interval was <0.4, and (3) a logistic regression model score was ≥0.518.

#### CUMC

SNVs were called on a per-trio basis using SAMtools (v1.3.1-42) and BCFtools (v1.3.1–174). We generated trio VCF files using samtools ‘*mpileup’* command with options *‘–q 20 –Q 13*’ corresponding to mapQ and baseQ thresholds of 20 and 13 respectively, followed by bcftools ‘*call’* with option ‘*–p 1.1*’ to expand the set of variant positions to be evaluated for mosaicism. In contrast to the OHSU pipeline, BAQ was used to potentially reduce false positive SNV calls caused by misalignments.^76^ To identify de novo variants from trio VCF files, we selected for sites with (i) a minimum of six reads supporting the alternate allele in the proband and (ii) for parents, a minimum depth of ten reads and 0 alternate allele read support. Variants were then annotated using ANNOVAR (v2017-07-17) to include information from refGene, gnomAD (March 2017), 1000 Genomes (August 2015), ExAC, genomicSuperDups, COSMIC (v70), and dbSNP (v147) databases. CADD,^10^ MPC^11^ were used to annotate variant functional consequence.

#### Preprocessing and QC

To reduce the noise introduced by our variant calling approach, we preprocessed our variants using a set of filters. Since our method is allelic depth-dependent, we took a conservative filtering approach to reduce the impact of false positives on model parameter estimation. We first filtered our variant call set for rare heterozygous coding variants (MAF ≤ 1 × 10^−4^ across all populations represented in gnomAD and ExAC databases). To account for regions in the reference genome that are more challenging to resolve, we removed variant sites found in regions of nonunique mappability (score <1; 300 bp), likely segmental duplication (score >0.95), and known low complexity.^77^ We then excluded sites located in *MUC* and *HLA* genes and imposed a maximum variant read depth threshold of 500. To account for common technical artifacts, we used SAMtools PV4 *p*-values with a threshold of 1 × 10^−3^ to exclude sites with evidence of baseQ bias, mapQ bias, and tail distance bias. To account for potential strand bias, we used an in-house script to flag sites that have either (1) 0 alternate allele read support on either the forward or reverse strand or (2) *p* < 1 × 10^−3^ and OR < 0.33 or OR > 3 when applying Fisher’s method to compare strand based reference or alternative allele counts. Finally, we excluded sites with frequency >1% in the SPARK pilot, as well as sites belonging to outlier samples (with abnormally high de novo SNV counts, cutoff = 7) and complex variants (defined as sites with neighboring de novo SNVs within 10 bp).

#### IGV visualization of low allele fraction de novo SNVs

To identify likely false positives among our low allele fraction (VAF <0.3) de novo SNVs, we used Integrative Genomics Viewer (IGV v2.3.97) to visualize the local read pileup at each variant across all members of a given trio. We focused on the allele fraction range 0.0–0.3 since this range captures the majority of the technical artifacts that will negatively impact downstream parameter estimation. Sites were filtered out if (1) there were inconsistent mismatches in the reads supporting the mosaic allele, (2) the site overlapped or was adjacent to an indel, (3) the site had low MAPQ or was not primary alignment, (4) there was evidence of technical bias (strand, read position, tail distance), or (5) the site was mainly supported by soft-clipped reads.

#### Empirical bayes postzygotic mutation detection model

To distinguish variant sites that show evidence of mosaicism from germline heterozygous sites, we modeled the number of reads supporting the variant allele (*N*_*alt*_) as a function of the total site depth (*N*). In the typical case, *N*_*alt*_ follows a binomial model with parameters *N* = site depth and *p-value* *=* mean VAF. However, we observed notable overdispersion^78,79^ in the distribution of variant allele fraction compared with the expectations under this binomial model. To account for this overdispersion, we instead modeled *N*_*alt*_ using a beta-binomial distribution. We estimated an overdispersion parameter *θ* for our model whereby for site depth values *N* in the range 1–500, we (1) bin variants by identifying all sites with depth *N*, (2) calculate a maximum-likelihood estimate *θ* value using *N* and all *N*_*alt*_ values for variants in a given bin, and (3) estimate a global *θ* value by taking the average of *θ* values across all bins, weighted by the number of variants in each bin.

We used an expectation-maximization (EM) algorithm to jointly estimate the fraction of mosaics among apparent de novo mutations and the FDR of candidate mosaics. This initial mosaic fraction estimate gives a prior probability of mosaicism independent of sequencing depth or variant caller and allows us to calculate, for each variant in our input set, the posterior odds that a given site is mosaic rather than germline.

##### Finalized union mosaic call set and validation selection

The high confidence call sets from the two parallel mosaic determination approaches were combined, and all candidate mosaic variants were then inspected manually in IGV. Variants in regions with multiple mismatches or poor mapping quality were removed, and the remaining mosaics comprised the high confidence mosaic call set. For calls that were unique to one approach, the variant was annotated with which quality filter it initially failed. Variants that were flagged as low confidence germline by CUMC approach but mosaic by OHSU approach had posterior odds >1 and were thus retained in the union call set.

##### CNV detection

De novo and rare inherited CNVs were independently called by two centers—UW and SF. The final CNV list included all autosomal CNVs that were called by both SF and UW pipelines either with reciprocal overlap of at least 50% or when the CNV from one pipeline was completely within the CNV from the other pipeline. In both cases, the overlapping region was reported as the final region and annotated as described below. CNVs called only by one pipeline were considered as high confidence CNVs if they were called by at least two tools or if they were de novo CNVs confirmed by manual inspection of plots on exome data. High confidence CNVs were also included in the final list after discussion and manual inspection of plots on exome data. De novo CNVs were additionally inspected on BAF and LRR plots on genotyping data. CNVs that had at least 75% overlap with known segmental duplications (segDups track for hg19 from UCSC browser) were excluded. All CNVs were annotated with the list of RefSeq HG19 genes, OMIM genes, brain embryonically expressed genes,^3^ brain critical genes,^19^ ASD significant,^80^ and ASD related genes^8,14^ that have their coding regions overlapping with the CNV. CNVs greater than or equal to 50 kbp in size were annotated with morbidity map^81^ case and control frequencies using a 50% reciprocal overlap while CNVs < 50 kbp were annotated with their frequency in the 1000 genomes project^82^ using a 50% reciprocal overlap. We do note that it is possible some events may be missed with this annotation because of different platforms (e.g. exome, array, and genome), but the two analyses provide reasonable insight into the population prevalence of large and smaller CNVs in the general population. In addition, each found gene was annotated with pLI (ExAC release 0.3, http://exac.broadinstitute.org/downloads), ASD,^83^ RVIS,^84^ LGD,^85^ and SFARI Gene scores (version q1, 2018, https://gene.sfari.org/database/gene-scoring/). dnCNVs that affect DUSP22 and olfactory genes were excluded due to high variability in copy number of those regions among individuals.^86^

#### UW, detection using XHMM and CoNIFER

CNVs from WES were called using CoNIFER and^87^ XHMM.^88^ CoNIFER version v0.2.2 was used with the *S* value, –svd 7, set as a threshold as suggested by the scree plot. XHMM version statgen-xhmm-3c57d886bc96 was used with the following parameters –minTargetSize 10 –maxTargetSize 10000 –minMeanTargetRD 10 –maxMeanTargetRD 500 –minMeanSampleRD 25 –maxMeanSampleRD 200 –maxSdSampleRD 150 to filter samples and targets, and then to mean-center the targets; PVE_mean –PVE_mean_factor 0.7 was used to normalize mean-centered data using PCA information; –maxSdTargetRD 30 was used to filter and *z*-score centers (by sample) the PCA normalized data; and then to discover CNVs in all samples. Calls from CoNIFER and XHMM were merged in a VCF file using https://github.com/zeeev/mergeSVcallers with the following parameters -t xhmm,conifer -r 0.5 -s 50000, then merged VCF was sorted by Picard version v2.5.0, and zipped and indexed with Tabix version v0.2.6. We re-genotyped each XHMM and CoNIFER CNV event by assessing the RPKM values from the CoNIFER workflow on an individual. Probands were considered to have a deletion if their average RPKM value was less than −1.5 s.d and have a duplication if their average RPKM value was greater than 1.5 s.d. For an event to be considered as variant in a parent, we required an average ZRPKM less than −1.3 or greater than 1.3 for deletions and duplications, respectively.

#### UW, CNV validation using SNP microarray

We generated an independent CNV callset for validation purpose using SNP microarray genotyping data generated from Illumina InfiniumCoreExome-24_v1.1, where IDATs (*n* = 1,421) were processed using Illumina Genome Studio Software. CNV analysis was performed using the Illumina CNVpartition algorithm version v3.2.0. Log R Ratio data for all samples and probes was exported. PennCNV^89^ version v1.0.4 was used to detect CNVs with the following parameters -test –hmm -pfb all.pfb –gcmodelfile –confidence. We determined the maximum and minimum overlap of SNP microarray CNVs based on the presence of WES probes to make the array calls more similar to the exome calls and considered an event to have support by PennCNV or CNVpartition if there was at least 50% reciprocal overlap. We also generated per probe copy number estimates using CRLMM^90,91^ version 1.38.0 as previously described^14^ and genotyped each candidate WES CNV. Deletions were considered variant if they had a *p*-value less than 0.05 and a mean percentile rank less than 30. Duplications were considered variant if they had a *p*-value less than 0.05 and a percentile rank of mean greater than 70. CNVs passing the RPKM genotyping were combined with the CNV data from CRLMM, PennCNV, and CNVPartition. We considered WES CNVs as valid if there was support for gain or loss from the PennCNV, CNVpartition, or CRLMM approaches described above. We assessed inheritance using both SNP and WES data and preferentially scored inherited events over de novo CNVs.

#### SF

CNVs were called with two tools - xHMM v 1.0^92^ and CLAMMS v 1.1.^93^ xHMM**:** CNVs were called with default settings (except not filtering on the maximum target size), including filtering low complexity and GC extreme targets. CLAMMS: CNVs were called with INSERT_SIZE = 390 bp and training per-sample-models on sample specific reference panels due to the observed batch effect in the data; CLAMMS calls were filtered for all CNVs with Q_EXACT less than 0, or Q_SOME less than 100, or CNVs that were in samples with more than 70 predicted CNVs of the size at least 10 Kb and of quality score Q_SCORE at least 300. The inheritance status of the autosomal CNVs was determined by default xHMM protocol for de novo CNVs identification with plink 1.07^94^ and Plink/Seq 0.10 [https://atgu.mgh.harvard.edu/plinkseq/]. Similar protocol was implemented in java for CLAMMS analysis. For each tool, two tiers of CNV calls—the most confident calls (tier 1) and less confident calls (tier 2)—were defined, based on de novo and transmission rates for different cuts on quality scores: SQ (phred-scaled quality of some CNV event in the interval) and NQ (phred-scaled quality of not being diploid, i.e., DEL or DUP event in the interval) in xHMM and Q_SOME (phred-scaled quality of any CNV being in this interval) in CLAMMS. xHMM tier1 included all autosomal CNVs with both SQ and NQ quality scores of at least 60, and tier2—all autosomal CNV calls with quality scores between 30 and 60. Samples with more than 10 de novo CNVs in xHMM tier1 of size at least 10 kb were excluded. CLAMMS tier1 included all predictions with quality score 999, except predictions for 25 probands that have CNVs of size greater than 500 kb with quality score 999 or predictions, which region was partially inherited and partially de novo; tier 2 included those excluded from tier 1 predictions as well as all CNVs with quality score Q_SOME at least 400 and less than 999. Predictions by both methods that had less than 3 exons or at least 75% overlap with known segmental duplications (segDups track for hg19 from UCSC browser) were removed from the list. The final list of CNV predictions included all CNVs from tier 1 predicted by either xHMM or CLAMMS and “intersection” of tier 2 sets from both tools, that is, CNVs that were confirmed by two tools with reciprocal or cumulative reciprocal overlap of at least 50%. In the latter case, CNV predicted by one tool is covered by a set of CNVs predicted by the other tool. If a CNV from xHMM or CLAMMS was confirmed by the other tool, the overlapping region was reported as the final region. CNVs were removed from the analysis if it had more than half of its length overlapping with the ACMG secondary findings v2 gene^74^ (except *PTEN, TSC1*, and *TSC2*). If such gene covers less than 50% of CNV, the part of CNV without the gene was kept if it has at least 25% of its length not covered by segmental duplications. To identify higher confidence CNV predictions, xHMM and CLAMMS plots were manually investigated for each CNV in the final SF list. In addition, SF predictions were compared with PennCNV^89,95,96^ calls from array data, which have confidence score of at least 100. All reciprocal overlaps of at least 50% were treated as additional evidence for CNV support.

#### UW, chromosome aneuploidy assessment

We also assessed evidence of chromosomal aneuploidy by calculating sequence read depth using SAMTools^10^ version 1.4 on a per chromosome basis normalizing by the relative density of WES probes and comparing the normalized value for each chromosome to the normalized value on chromosome 1 (assumed to be diploid). For autosomes, we multiplied this number by two to get the estimate of chromosomal copy number. We did not multiply by two for the X or Y chromosomes. To further assess the chromosomal copy number, the heterozygosity was calculated for all SNPs and indels. For heterozygous sites, the absolute mean deviation from 0.5 was also calculated. We assessed both metrics to identify outliers. Aneuploidies were required to have support from both the read depth and SNP/indel metrics.

### Burden of de novo variants

Baseline mutation rates for different classes of de novo variants in each GENCODE coding gene were calculated using a previously described mutation model.^9^ Briefly, the trinucleotide sequencec context was used to determine the probability of each base mutating to each other possible base. Then the mutation rate of each functional class of point mutations in a gene was calculated by adding up the mutation rate of each nucleotide in the longest transcript. The rate of frameshift indels was presumed to be 1.1 times the rate of nonsense point mutations. The expected number of variants in different gene sets were calculated by summing up the class-specific variant rate in each gene in the gene set multiplied by twice the number of patients (and if on chromosome X, further adjusted for female-to-male ratio^97^).

The observed number of variants in each gene set and case group was then compared with the baseline expectation using a Poisson test. In all analyses, constrained genes were defined by a pLI score of ≥0.5. To compare with previously published ASD studies, we collected published de novo variants identified in 4773 simplex trios from three largest ASD studies to date.^3,4,7^ To account for platform differences, the baseline mutation rate of each gene was scaled so that the exome-wide expected number of silent variants matches the observed count.

### TADA analysis

To perform TADA analysis of de novo variants, we assumed the fraction of disease genes is 5% as estimated by previous studies.^26,98^ The prior relative risk for LGD variants and D-mis (defined by CADD > = 25) were specified as Gamma (18,1) and Gamma (6,1). The prior mean relative risks were determined using the relationship between burden and relative risk as described previously.^26^ The baseline mutation rate of each gene was the same as used in burden analysis. The analysis was performed on de novo variants of 4773 published trios and after combing de novo variants identified from SPARK pilot trios.

### Laminal layer and cell type enrichment

To evaluate the expression specificity of laminal layer of human developing cortex, we analyzed RNA-seq data of neocortical samples of BrainSpan^48^ following the method of Parikshak et al.^45^ The expression specificity was measured by a *t*-statistic comparing the expression level in each layer against all other layers. Two candidate ASD risk genes (*PAX5, KDM1B*) were not included in the analysis due to the low expression levels (RPKM <1 for at least 20% available neocortical samples). To evaluate cell-type specificity, we used published data of mouse neuronal cell types inferred from analyzing single cell RNA-seq data of fetal and adult mouse brains generated by the Karolinska Institutet (KI),^99^ and human CNS cell types inferred from a single nucleus RNA-seq data.^50^ The mouse orthologs of human genes were retrieved from MGI database.^100^ The cell-type specificity was measured by a specificity index which is the mean expression level in one cell type over the summation of mean expression level across all cell types.^101^ To analyze the overall trend of specificity of a gene set, the mean specificity measure of its genes was compared with 10,000 sets of randomly drawn genes matched for the transcript length and GC content and the enrichment is measured by the standard deviation from the mean specificity of random gene sets.^101^

### Network and functional analysis

The network depicted in Fig. 2a was constructed using the top decile of forecASD genes, SFARI Genes scoring 1 or 2, and SPARK newly implicated genes (6 in total). These genes were projected onto the STRING network^102^ (v10) using the igraph R package (1708 genes). Edges within the STRING network were thresholded at 0.4, according to the authors’ recommendation. The largest connected subcomponent (1664 genes) was then extracted as the basis for further network analysis. Clustering was performed on the fully connected network using the fastgreedy community function available within the igraph package. Clusters with fewer than 30 genes were not considered for further analysis (none of these clusters contained the six genes highlighted here). Following the first round of clustering, clusters with >150 genes were subject to an additional round of clustering, with the goal of separating broad functions of genes into more specific subcomponents. This process resulted in ten clusters. Each cluster was assessed for functional enrichment using the Gene Ontology^103^ as accessed through the clusterProfiler package within R. During the functional analysis the background gene universe was always set to the full set of genes represented among the ten clusters. Visualization of this network analysis was performed in Cytoscape.^104^ The top five most significant GO terms associated with each cluster are available in the Supplementary Data 9. Cluster labels in Fig. 2 were chosen as the most representative among the top terms for each cluster. Figure 2b was constructed using the subset of the larger network (Fig. 2a), corresponding to SPARK newly implicated genes and SFARI Gene genes scoring 1 or 2 (88 genes). These genes were projected onto the STRING network within Cytoscape using the STRINGapp. All nonzero-weighted edges were considered. The fully connected component was visualized, which resulted in two genes being dropped (*DEAF1* and *RANBP17*). Edges adjacent to newly implicated genes with a STRING interaction score of ≥0.4 are highlighted.

#### ForecASD analysis

We used a recently developed method, forecASD^40^ that indexes support for a gene being related to ASD by integrating genetic, expression, and network evidence through machine learning. We examined the forecASD scores of candidate ASD risk genes from the TADA analysis and compared them to the remainder of the genome using a Wilcoxon rank-sum test. We similarly used the Wilcoxon test and employed two predictive features used by forecASD (BrainSpan_score and STRING_score) to assess whether the new genes showed similarity to known ASD risk genes in terms of brain expression patterns and network connectivity. Importantly, because forecASD uses previously published TADA scores among its predictive features, which are strongly correlated with updated TADA scores, we investigated whether the elevated forecASD scores in our candidate genes could be explained solely by the previous TADA scores. Specifically, we fit a logistic regression model with the candidate ASD risk genes labeled as ‘1’ and 500 size-matched background genes (not listed in the SFARI gene database) labeled as ‘0’ in the dependent variable (Y). Separate models were fit using either forecASD or TADA^8^ scores as predictors, or both together in a full model. Both TADA and forecASD were significantly associated with the “new gene” indicator when considered in isolation (*P* <0.001 for both, *Z*-test on logistic regression coefficients). However, when included together in a model of Y, forecASD remained significantly associated (*p*-value = 0.00012, *Z*-test on logistic regression coefficients) while TADA lost significance (*p*-value = 0.41, *Z*-test on logistic regression coefficients). The Akaike information criterion (AIC) indicated that the forecASD-only model was a more optimal fit compared with either the TADA-only or TADA + forecASD fit. This analysis suggests that the elevated forecASD scores observed in the ten new genes cannot be fully explained by the use of TADA as a predictor in forecASD.

### Reporting summary

Further information on research design is available in the Nature Research Reporting Summary linked to this article.

## Supplementary information

Supplementary Information

Reporting Summary

Data Set 1

Data Set 2

Data Set 3

Data Set 4

Data Set 5

Data Set 6

Data Set 7

Data Set 8

Data Set 9

Data Set 10

## Data Availability

Methods for SNV, Indels, CNV analysis are available at https://genomicpipelines.sparkforautism.org/.

## Supplementary information

**Supplementary information** accompanies the paper on the *npj Genomic Medicine* website (10.1038/s41525-019-0093-8).

## Data availability

The genomic and phenotypic data for the 1379 individuals analyzed in this study is available by request from SFARIBase (https://www.sfari.org/resource/sfari-base/) with accession ID: SFARI_SPARK_WES_p.
