# Supplementary material for: Exome sequencing of 457 autism families recruited online provides evidence for autism risk genes
Source: NPJ Genom Med. 2019 Aug 23;4:19. doi: 10.1038/s41525-019-0093-8 (PMC6707204; doi:10.1038/s41525-019-0093-8)
Supplement: Supplementary file 1 — Supplementary Information [file 41525_2019_93_MOESM1_ESM.pdf]

Supplementary Information Table of Contents

Legends for Supplementary Data 1-10………………………….pp. 2-4

Supplementary Table 1……………………………………………p. 5

Supplementary Figures 1-14…………………………………pp. 6-19

Supplementary Data 1: All *de novo* SNV/indels in the affected offspring.

Supplementary Data 2: A) Burden of *de novo* variants in SPARK ASD trios and **B)** in published ASD trios. Likely gene disruptive (LGD) variants include frameshift indels, stop gain SNVs, and variants affecting canonical splice sites. Deleterious missense variants are defined by CADD score[10] ≥ 25 or by MPC score[105] ≥ 2. Genes are classified as constrained genes based on pLI≥0.5. The enrichment of observed de novo variants were compared to the baseline expectations[9] by one-sided Poisson test. Baseline mutation rates were recalibrated so that the observed number of de novo silent mutations matches the expectation.

Supplementary Data 3: All singleton LGD variants (transmitted or un-transmitted) in known ASD/NDD genes. Singleton variants are defined as appearing only once in the SPARK pilot cohort. Rare singletons variants are singletons with ExAC allele frequency (all populations) < 0.001. Private singleton variants are singletons that are also absent from 1000 genomes, ESP, and ExAC databases.

Supplementary Data 4: All rare, inherited CNVs in the affected offspring.

Supplementary Data 5: All rare, de novo CNVs in the affected offspring.

Supplementary Data 6: List of likely mosaic variants.

Supplementary Data 7: Results from the TADA meta-analysis of *de novo* variants from published simplex ASD trios (n=4,773) and SPARK pilot trios (n=457). Only genes with *de novo* LGD or D-mis (defined by CADD>=25) variants observed in SPARK pilot trios are shown.

Supplementary Data 8: Support for the six newly statistically significant ASD risk genes and candidate ASD risk genes identified by TADA meta-analysis. Known ASD genes are defined as SFARIgene[108] score ≤ 2 or identified in a previous TADA meta-analysis (FDR<0.1)[8]). Known NDD genes were defined as those in the DDG2P database[16]. We systematically evaluated

constrained genes (pLI>=0.5) in which we identified de novo LGD variants in the SPARK cohort for which there are previously identified LGD variants or CNVs in individuals with developmental disorders and evaluated constrained genes (pLI>=0.5) disrupted by deletions that we identified in SPARK, which affect less than five constrained genes and overlap with previously published copy-number deletions. For each gene, we checked membership in the following gene sets that were previously associated with ASD: FMRP targets: genes whose mRNA translation in neurons is likely regulated by the FMR1 protein, based on bioinformatics prediction and regulatory sequence motifs[35]; PSD: post-synaptic density components based on human neocortex proteomics[36]; Embryonic: genes whose expression levels are high in post-mortem embryonic brains and then decrease after birth, based on BrainSpan expression data and computationally derived by Iossifov et al. 2014[3]; M2,M3,M16,M13: Gene co-expression modules that are enriched for known ASD genes from a previous analysis of Parikshak et al 2013[45]; Brain specific expression: genes specifically expressed in fetal or adult brain, defined as expression index for the fetal or adult brain greater than the median expression for the entire data-set and greater than twice the median expression of non-brain tissue; based on the Novartis Tissue Expression Atlas and previously compiled by Yuen et al. 2015[5]; Brain high expression: genes that have log2 (RPKM) >= 4.86 and at least 5 BrainSpan data points, compiled by Yuen et al. 2015[5]; Transcript regulation: GO:0006355; Chromatin modifier: GO:0016569; Nervous system development: GO:0007399; Nerve Impulse: GO:0019227 (neuronal action potential propagation), GO:0019226 (transmission of nerve impulse), and GO:0050890 (cognition) and Neuron projection: GO:0043005. In addition, we also searched the literature for studies implicating the gene in central nervous system development. Genes were excluded from consideration if they were not supported by any line of evidence listed above.

Supplementary Data 9: Summary of functional enrichment of network clusters depicted in Figure 2a.

Supplementary Data 10: All pathogenic (returnable) and possibly ASD-associated genetic variants.

Power

Expected number of total positive and true positive findings at a range of sample sizes

SampSize	TotPos (FDR<0.1)	TruePos (FDR<0.1)	TotPos (FDR<0.2)	TruePos (FDR<0.2)	TotPos (FDR<0.3)	TruePos (FDR<0.3)
1000	9.73	8.9	19.28	15.66	29.08	20.45
2000	32.33	29.43	56.27	45.7	81.94	58.05
3000	59.77	53.83	98.73	79.12	141.17	98.47
4000	88.63	80.13	140.6	113.24	198.38	138.87
5000	120.91	108.48	183.92	146.97	255.46	178.56
6000	150.92	135.37	223.88	179.18	307.39	215.05
10000	271.73	244.49	375.54	300.51	496.52	347.03
15000	389.93	350.56	515.07	411.14	660.86	461.56
20000	482.19	433.68	618.62	494.44	777.24	543.56
25000	563.47	507.98	708.66	568.37	877.13	615.02
30000	629.08	566.31	779.62	625.11	953.57	668.67
35000	669.5	603.32	820.36	656.85	994.9	697.06
40000	718.9	646.5	872.4	697.1	1049.6	734.5
45000	747.2	672.7	900.7	720.2	1077.7	754.2
50000	781.3	703.5	934.6	748.2	1112.2	778.2

Supplementary Table 1*:* Statistical power of TADA analysis. To simulate mutation data across all protein coding genes, we first randomly assigned each gene as ASD gene with probability of 0.05. Then for each ASD gene, we sampled relative risk (RR_i) for LGD and D-mis variants from prior distributions Gamma(18,1) and Gamma(6,1) which were the same as used in TADA analysis; for non-ASD gene, relative risk will be 1 for both types of variants. Then the number of observed de novo variants of class c for gene i will be sampled from Poission($2*N*u_{i,c}*RRi$), where $u_{i,c}$ is the baseline mutation rate and RRi is the relative risk. After generating the full data from all genes, we applied TADA to the dataset, and the procedure was repeated 100 times for each sample size. The table shows the average number of total positive findings and true positives at different FDR thresholds.

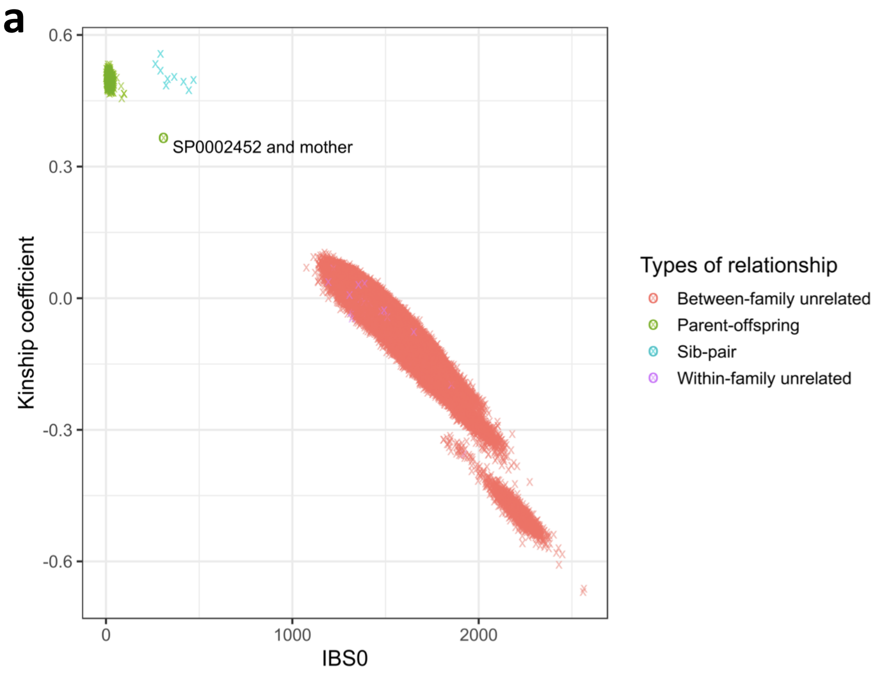

Supplementary Figure 1: Sample quality controls. **a**) Relatedness was verified based on the scatterplot of the estimated kinship coefficient and number of SNPs with zero shared alleles (IBS0). Parent-offspring, sibling pairs, and unrelated pairs can be distinguished as separate clusters on the scatterplot. One outlier parent-offspring pair (SP0002452 and mother) showed higher than expected IBS0 and was caused by parental chr6 iso-UPD. **b**) Sample sex was verified based on the ratio of heterozygous to homozygous genotypes on the X-chromosome, using normalized sequencing depth of X and Y chromosomes. Individuals with chromosomal abnormalities are highlighted.

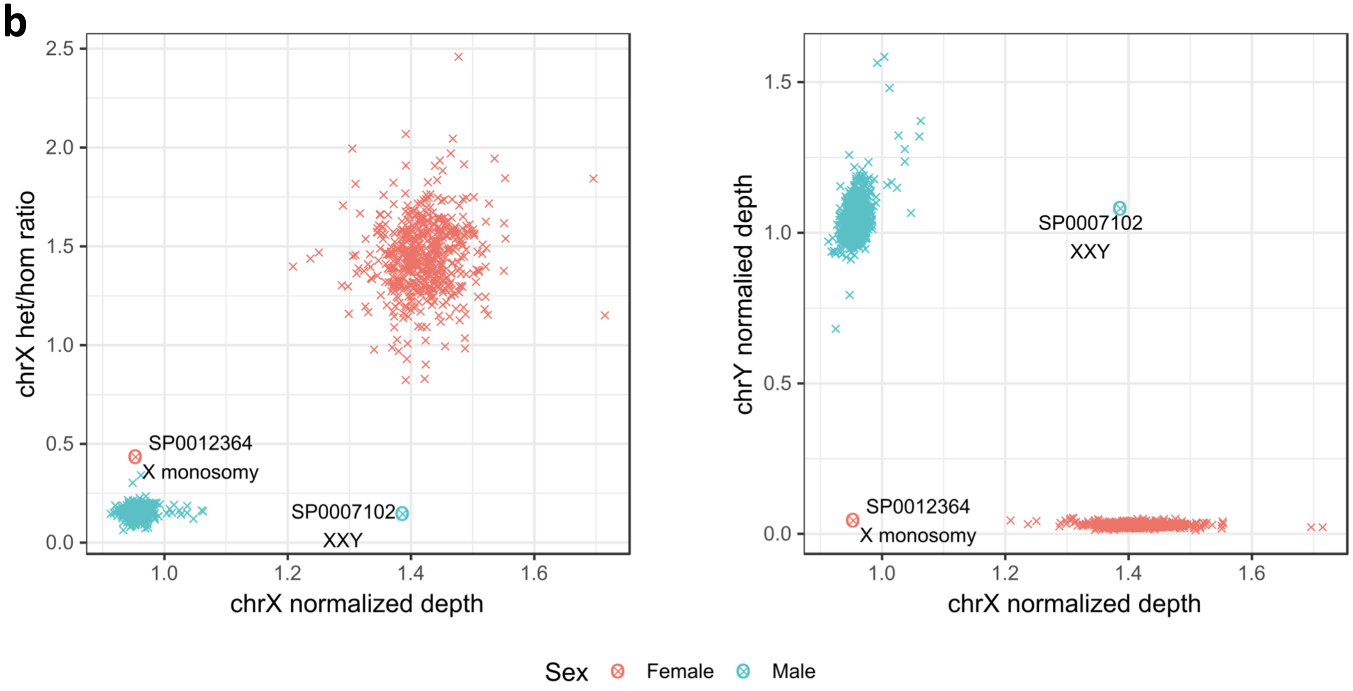

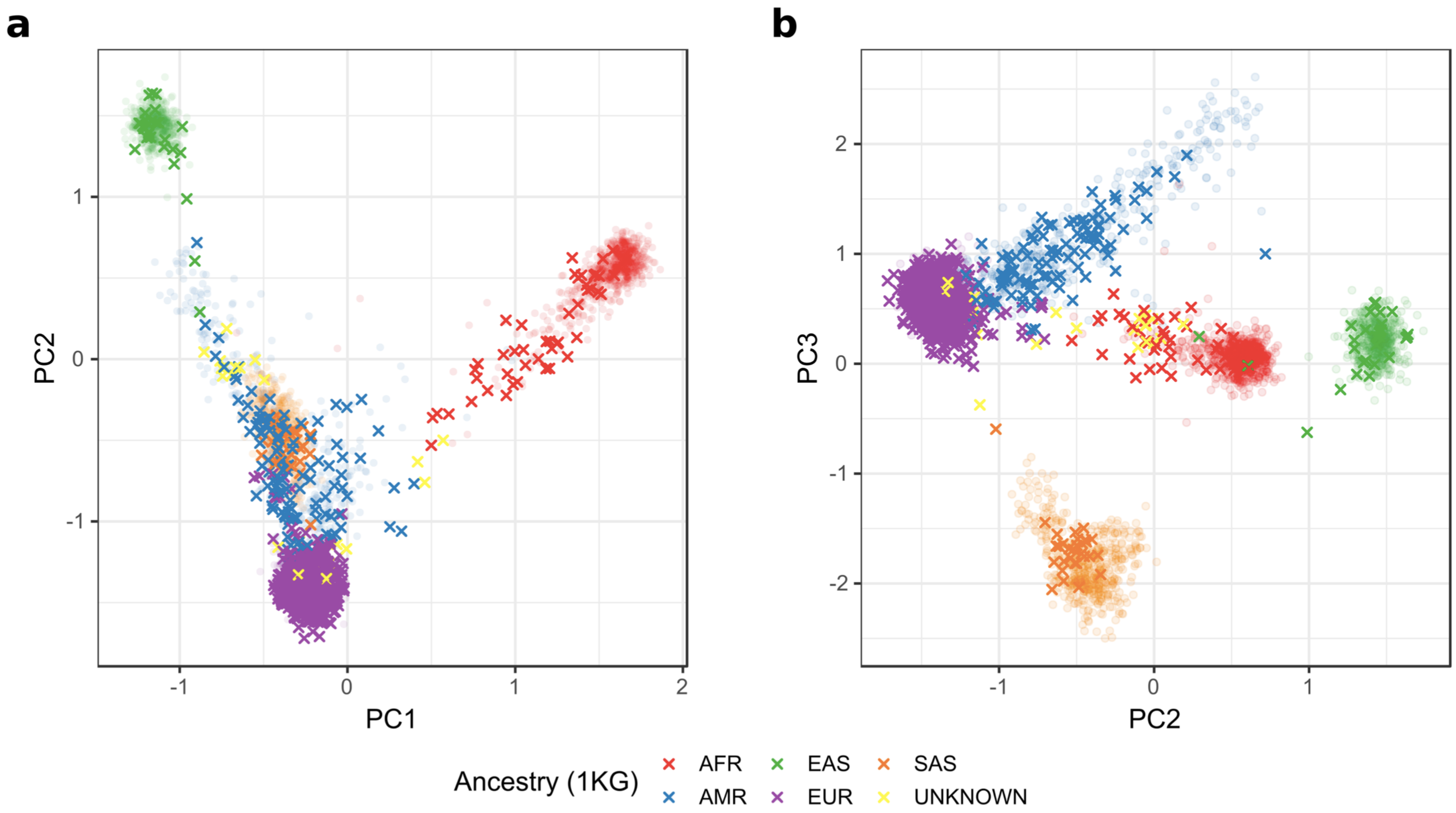

Supplementary Figure 2: Principal component (PC) analysis of sample ethnicity. Samples were projected onto the PC axes defined by the samples from 1000 Genomes Project (shown in light colors). **a**) The first two PCs can distinguish samples from three major continents. **b**) PC3 further distinguishes South Asians from Admixed Americans. Sample ethnicities were inferred based on the first four PCs using a machine learning approach implemented in peddy[103].

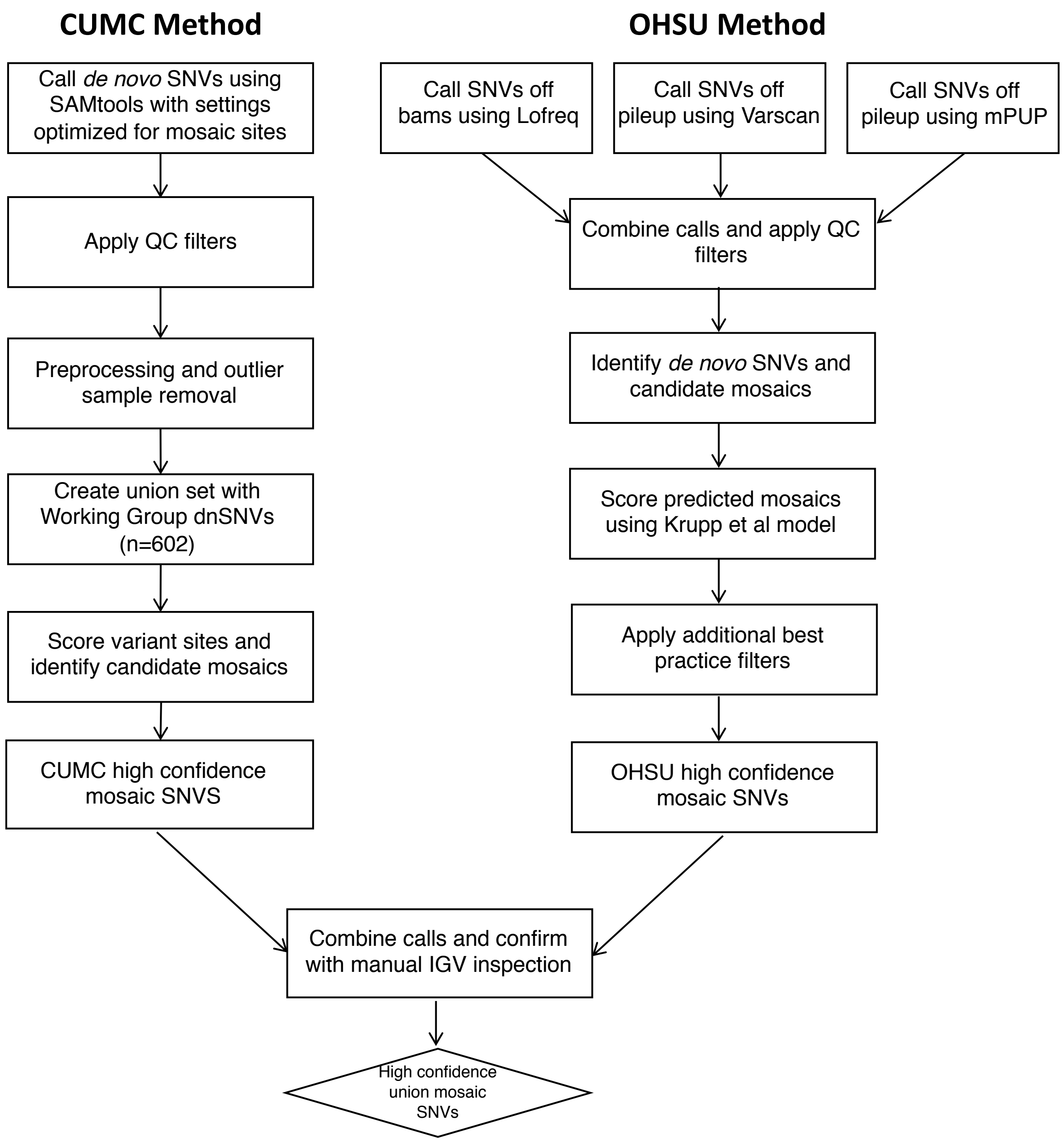

Supplementary Figure 3: Parallel calling approach for mosaic SNVs.

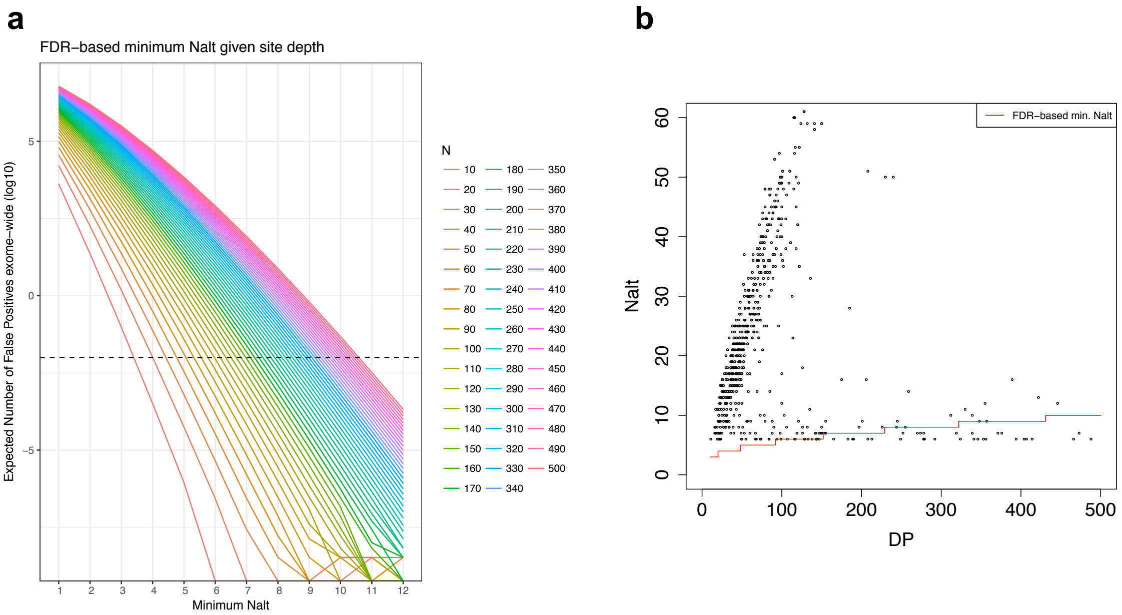

Supplementary Figure 4: Methods for FDR-based minimum alternate allele read depth (N_{alt}) threshold. Variants called by samtools are shown here. **a**) Theoretical FDR-based N_{alt} thresholds as a function of total read depth (N). Assuming that sequencing errors are independent and that errors occur with probability 0.005, with the probability of an allele-specific error being 0.005/3=0.00167, and given the total number of reads (N) supporting a variant site, we iterated over a range of possible N_{alt} values between 1 and 0.5*N and estimated the expected number of false positives due to sequencing error, exome-wide [$(1\text{-}Poisson(N_{alt}, \lambda=N*(0.00167))*3x10^7$]. Assuming one coding de novo SNV per individual[105] and that roughly 10% of de novo SNVs arise post-zygotically[21-22], we estimate there to be 0.1 mosaic mutations per exome. Under this assumption, to constrain theoretical FDR (in terms of distinguishing low allele fraction sites from technical artifacts) to 10%, we allowed a maximum of 0.01 false positives per exome. We used this cutoff to identify an FDR-based minimum N_{alt} threshold for each site as a function of total site depth. The dashed line denotes the threshold at which the expected number of false positives exome-wide is 0.01. **b**) FDR-based minimum N_{alt} threshold applied to samtools calls. Variant calls are plotted using total read depth (DP) and alternate allele read depth (N_{alt}). The red line marks the N_{alt} cutoff as a function of DP.

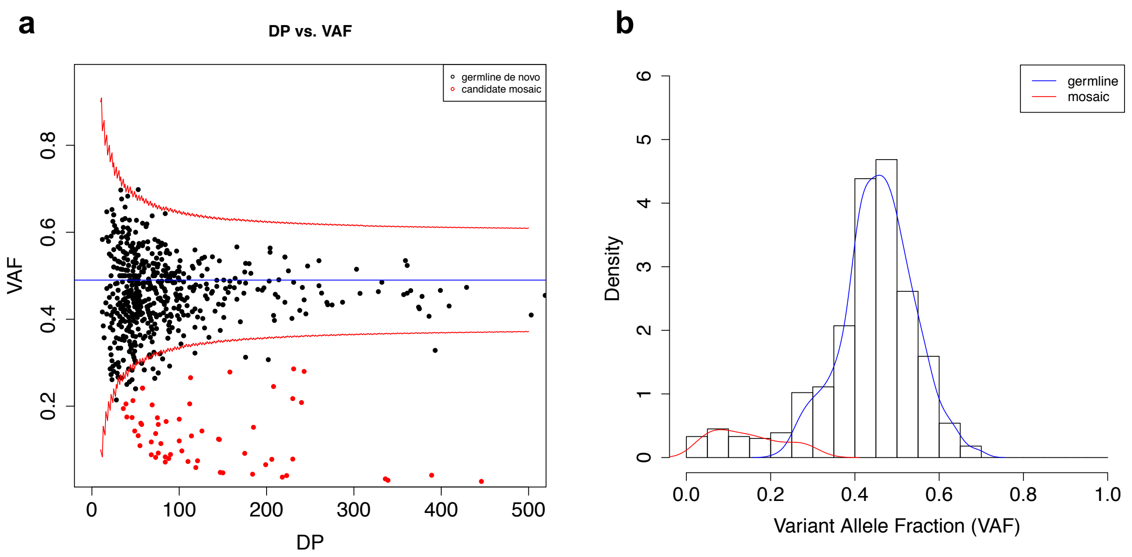

Supplementary Figure 5: Methods for mosaic candidate identification. Data shown are the consensus variant calls. **a**) Total read depth (DP) in relation to variant allele fraction (VAF). The blue line denotes the Beta-Binomial mean VAF and the red lines denote the 95% confidence interval. To calculate the posterior odds that a given variant arose post-zygotically, we first calculated a likelihood ratio (LR) using two models: M_0: germline heterozygous variant, and M_1: mosaic variant. Under our null model M_0, we calculated the probability of observing N_{alt} from a beta-binomial distribution with site depth N, observed mean germline VAF p, and overdispersion parameter θ. Under our alternate model M_1, we calculated the probability of observing N_{alt} from a beta-binomial distribution with site depth N, observed site VAF $p=N_{alt}/N$, and overdispersion parameter θ. Finally, for each variant, we calculated LR by using the ratio of probabilities under each model and posterior odds by multiplying LR by our EM estimated prior mosaic fraction estimate. Sites with posterior odds greater than 10 were predicted mosaic (corresponding to 9.1% FDR). **b**) Expectation-Maximization (EM) decomposition of variant allele fraction (VAF) into germline and mosaic distributions. Blue and red lines denote smoothed density curves for each distribution. We used an expectation-maximization (EM) algorithm to jointly estimate the fraction of mosaics among apparent *de novo* mutations and the false discovery rate of candidate mosaics. This initial mosaic fraction estimate gives a prior probability of mosaicism independent of sequencing depth or variant caller and allows us to calculate, for each variant in our input set, the posterior odds that a given site is mosaic rather than germline.

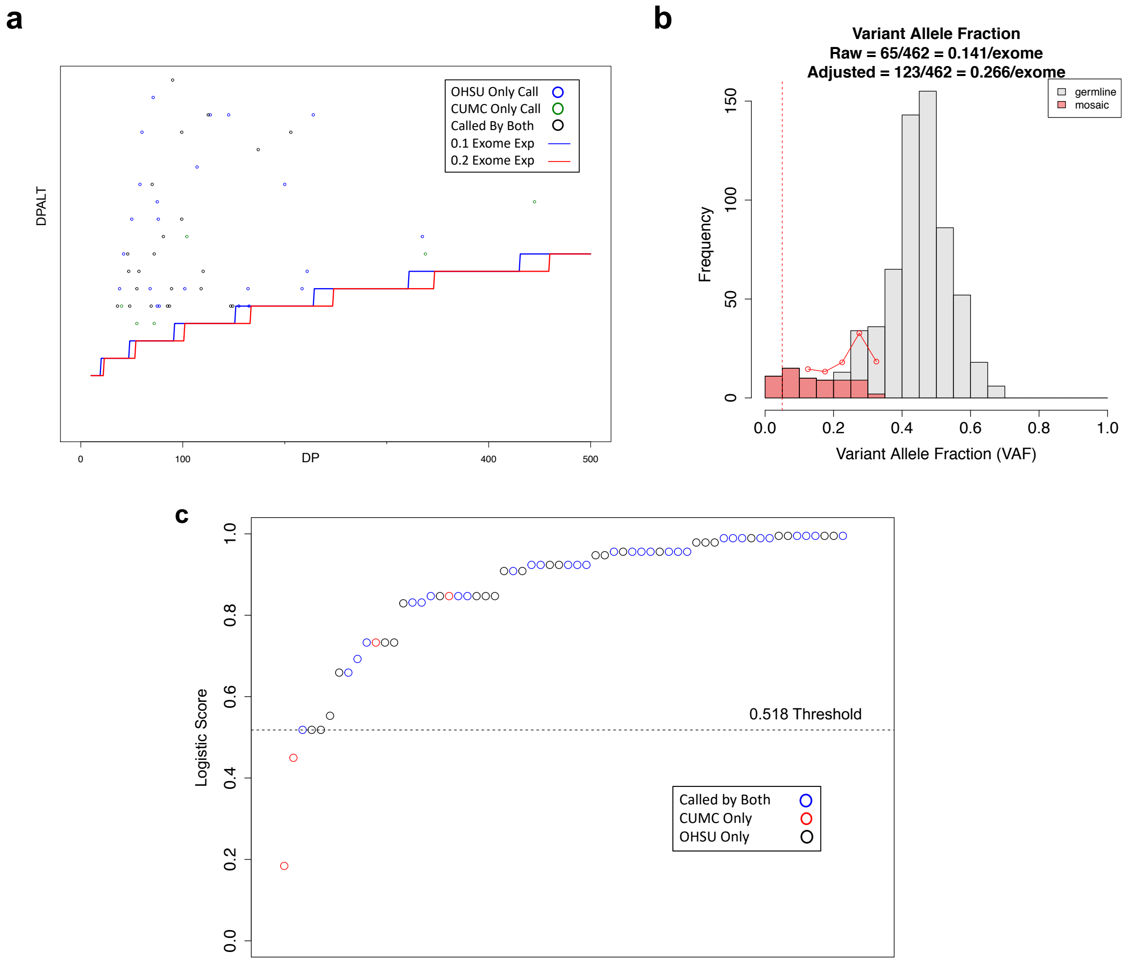

Supplementary Figure 6: Characterization of high confidence union mosaic calls. **a**) Alternative allele depth in relation to FDR based threshold. All calls are above the FDR threshold for both a 0.1 or 0.2 events per exome expectation. **b**) Variant allele fraction distribution. The grey and red bars denote germline and mosaic variants, respectively. The red line denotes the estimated true number of mosaics at each VAF window adjusted for mosaic detection power. Detection power is estimated as a function of variant allele fraction and sample average sequencing depth. The dashed vertical line denotes 5% VAF, below which estimated detection power is extremely limited and likely to artificially inflate adjusted counts. **c**) Percentile ranked distribution of Krupp et al.[22] logistic mosaic score, 0.518 was the applied threshold for OHSU pipeline. Scores are overall well distributed between overlapping and group specific calls. Three of the CUMC only calls were not scored as they were filtered out by the OHSU pipeline before scoring due to differences in segmental duplication annotation.

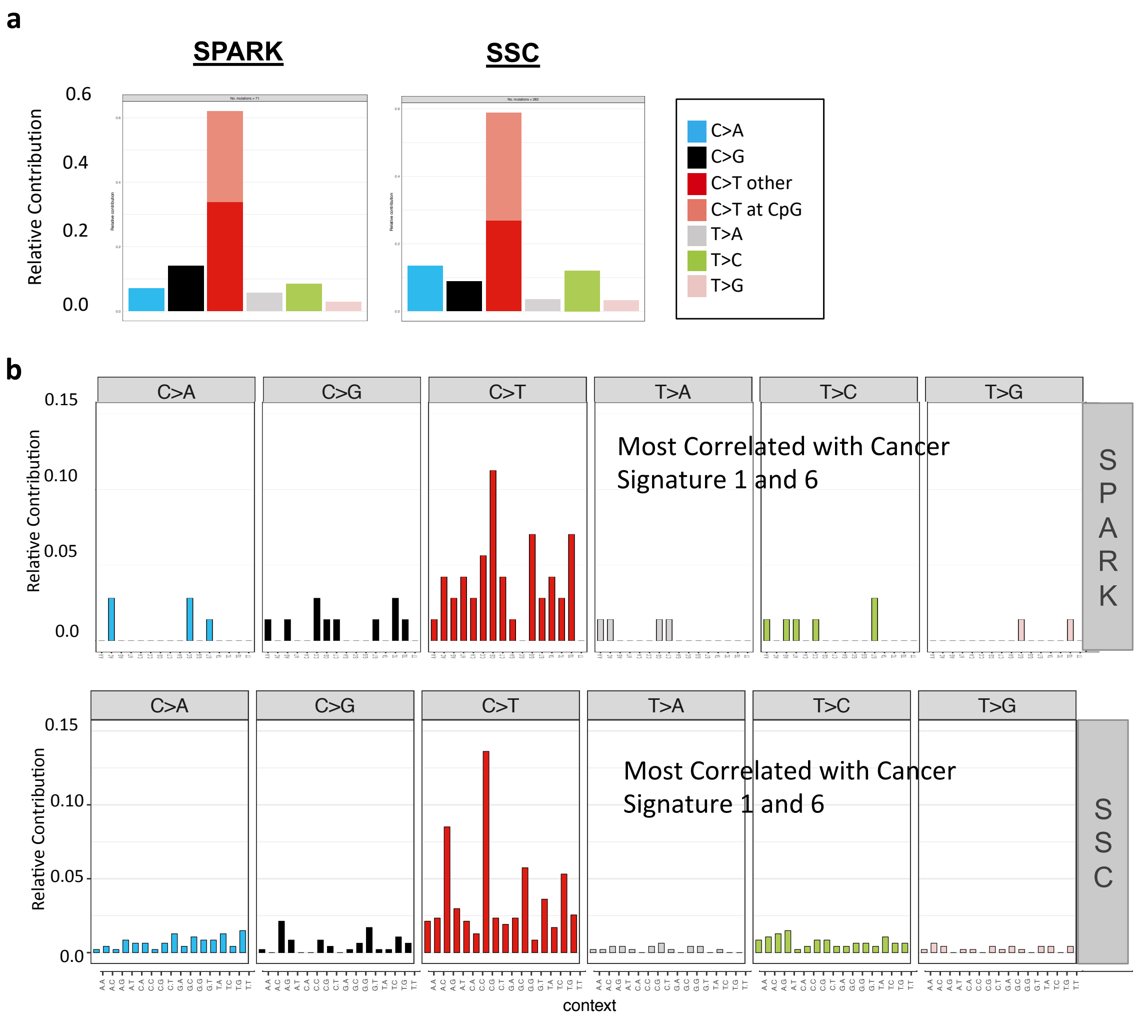

Supplementary Figure 7: Comparison of Mosaic Mutations Spectra and Signatures in SPARK and SSC. Mutational contexts and frequency were extracted and plotted using the R package *MuationalPatterns*[107]. **a**) Mutational spectrum of the six different possible substitutions for SSC and SPARK mosaic mutations. **b**) Mutational signature of the relative frequency of mutations (Y-axis) within trinucleotides (context) for SSC and SPARK mosaic mutations. Though there are fewer calls in SPARK due to the smaller cohort size, both SSC and SPARK show a strong correlation to the same Cancer Signatures which are indicative of endogenous and DNA mismatch repair mutational processes.

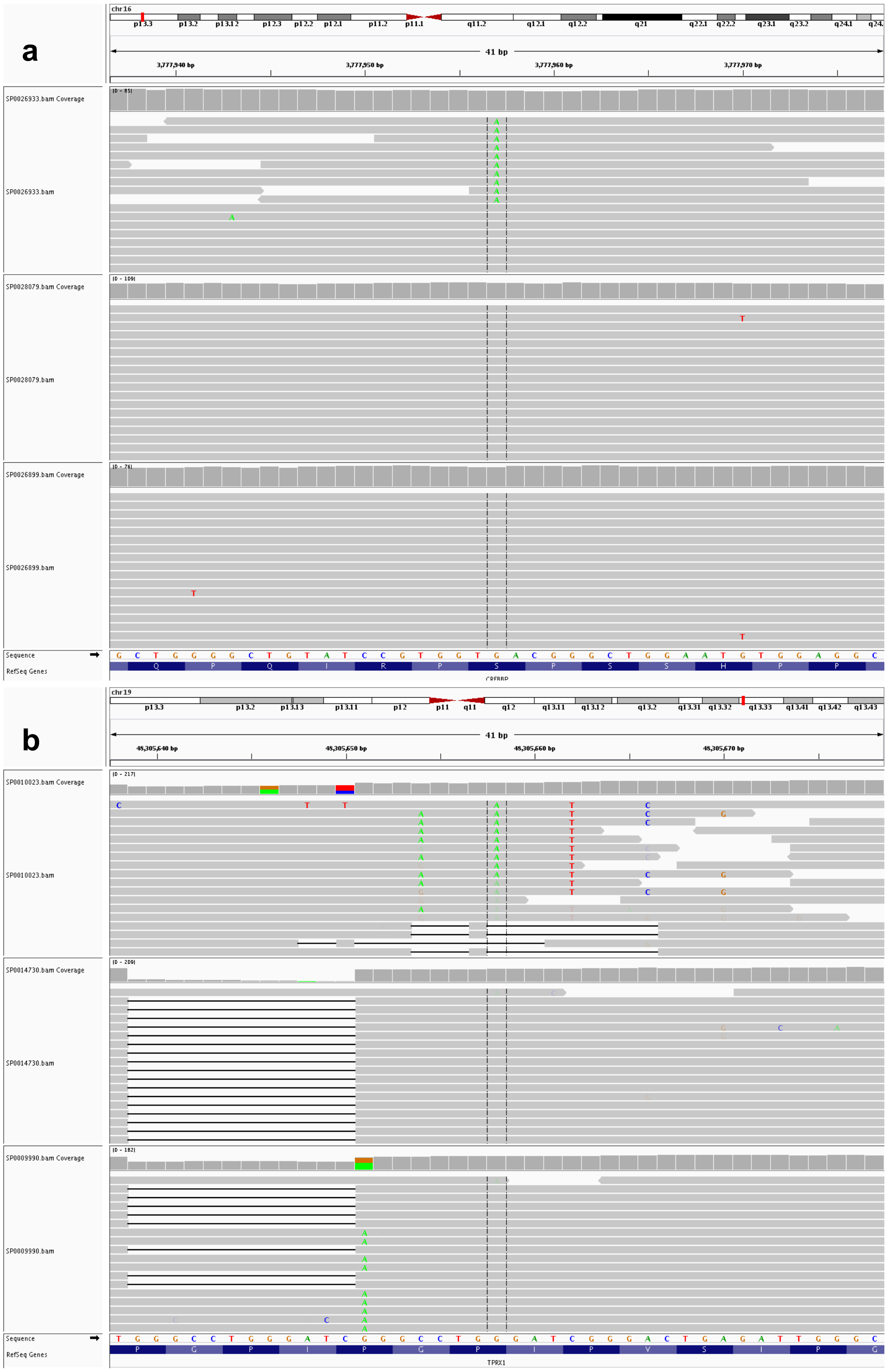

Supplementary Figure 8: IGV plots used in mosaic mutation visualization and review.
a) Example mosaic candidate passing IGV review – SP0026933:chr16:3777957:G>A
b) Example mosaic candidate failing IGV review –SP0010023:chr19:48305658:G>A

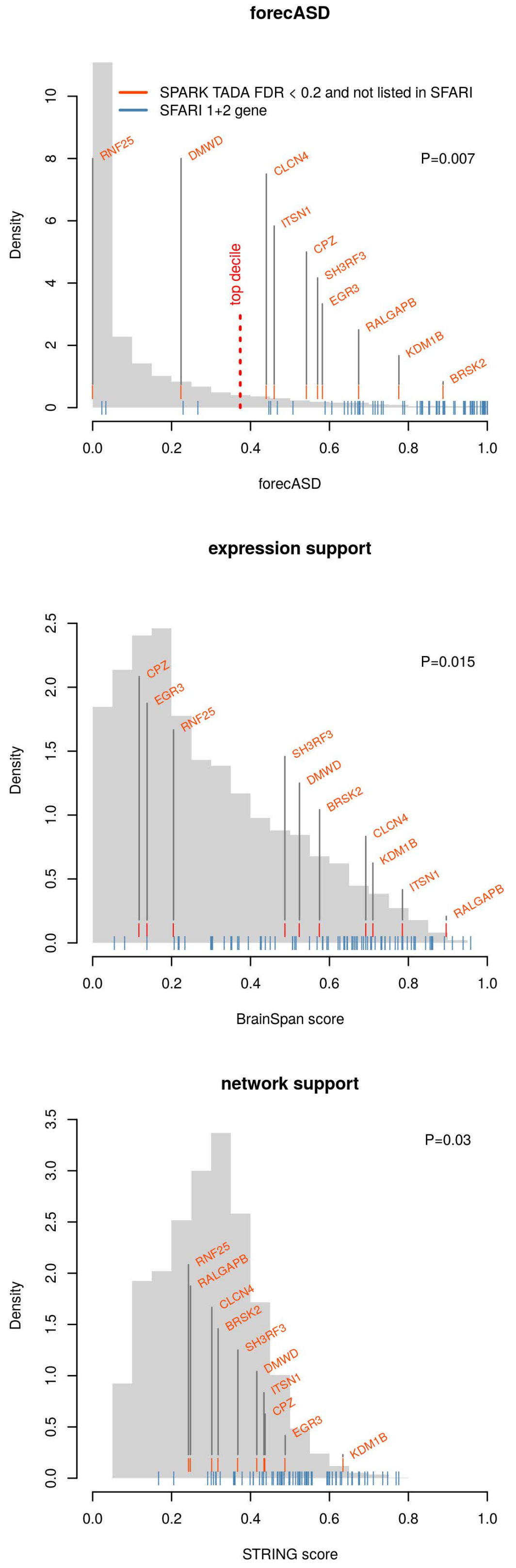

Supplementary Figure 9: Support for candidate ASD risk genes from forecASD. For this analysis, we considered genes that had a TADA FDR < 0.2 and were not listed in the SFARI Gene database: *BRSK2, KDM1B, RALGAPB, EGR3, SH3RF3, CPZ, ITSN1, CLCN4, RNF25*, and *DMWD*. These genes have significantly elevated forecASD scores (p-value=0.007, Z-test in logistic regression model with previous TADA scores as covariate), with 8 of the 10 genes in the top decile. Two constituent features in the forecASD ensemble (brain spatiotemporal expression and network topology) also show significantly elevated scores (p-value=0.015 and p-value=0.03, respectively,Wilcoxon test), suggesting that collectively, these genes show similar properties to known ASD genes beyond genetic association and across a diverse feature space, thereby supporting the robust biological plausibility of these genes. These associations are conservative estimates because they compare the distribution of evidence scores among the candidate genes described here to the remainder of the genome, which includes well-established ASD genes.

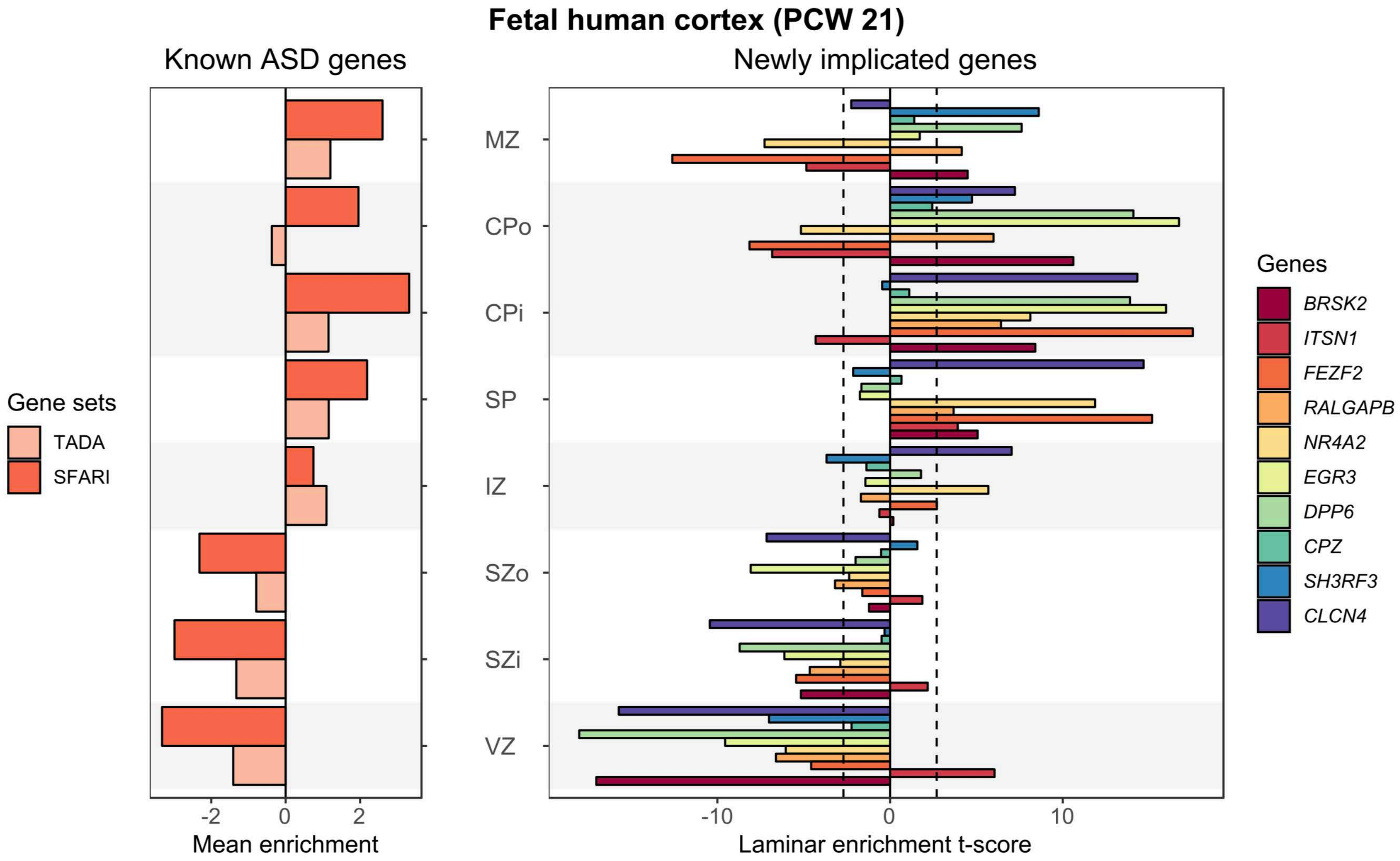

Supplementary Figure 10: Gene expression of candidate ASD risk genes in human fetal brain PCW21.

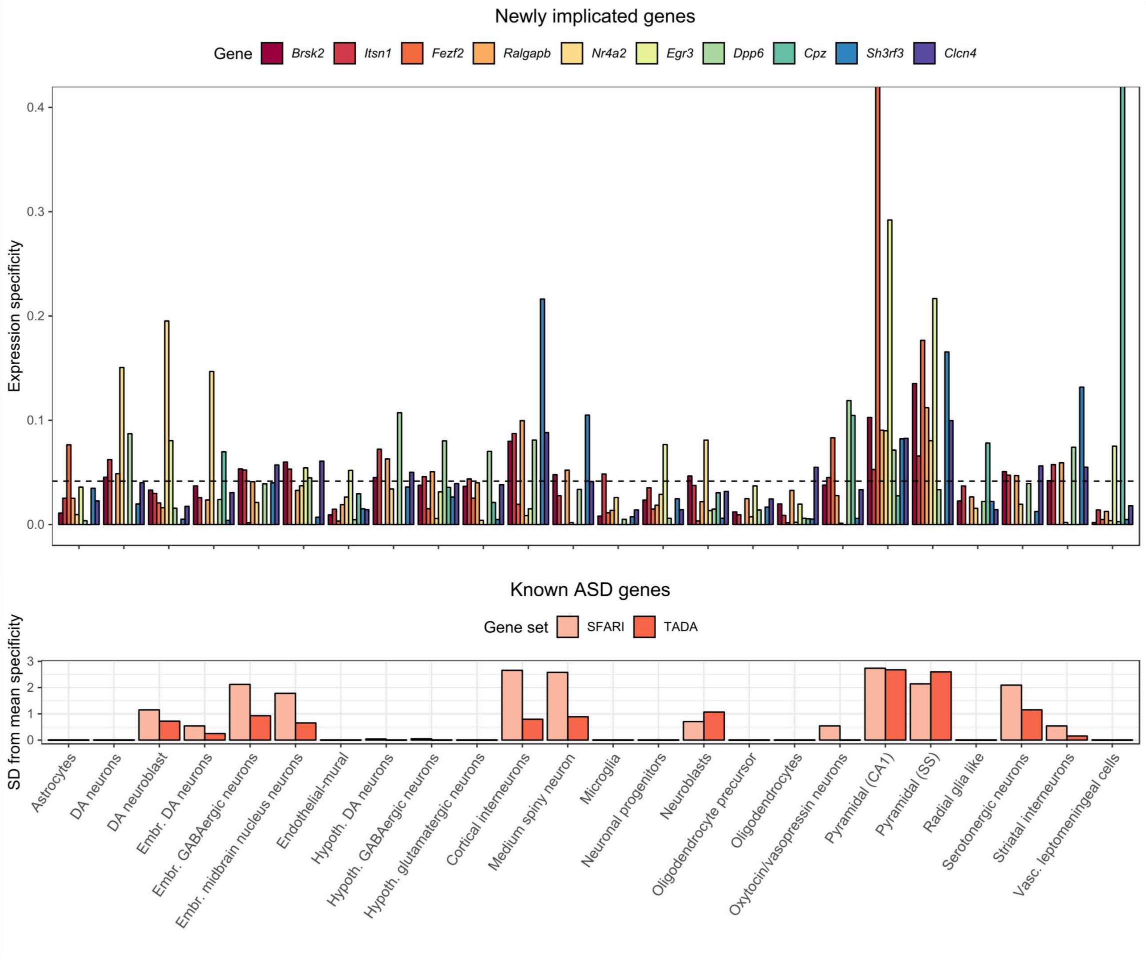

Supplementary Figure 11: Expression specificity of candidate ASD risk genes in single-cell RNA-seq data from fetal and adult mouse brains. The specificity of expression in a cell type is measured by a specificity index which is the mean expression level in one cell type over the summation of mean expression level across all cell types[100]. For a gene set, the mean expression specificity of its genes was compared with 10,000 sets of randomly drawn genes matched for the transcript length and GC content and the enrichment is measured by the standard deviation from the mean specificity of random gene sets[100]. The mouse neuronal cell types are defined by the analysis of single cell RNA-seq data of fetal and adult mouse brains generated by Karolinska Institutet (KI) and used in the previous study[49]. The mouse orthologs of human genes were retrieved from MGI database[99]. The known ASD genes show highest enrichment in pyramidal neurons (in hippocampus CA1 and somatosensory cortex), cortical interneurons, and medium spiny neurons. The first three enriched cell types were previously reported for the 65 ASD genes identified from TADA meta-analysis[100]. The newly implicated genes also show highest specificity in pyramidal neurons, suggesting functional convergence in these cell types.

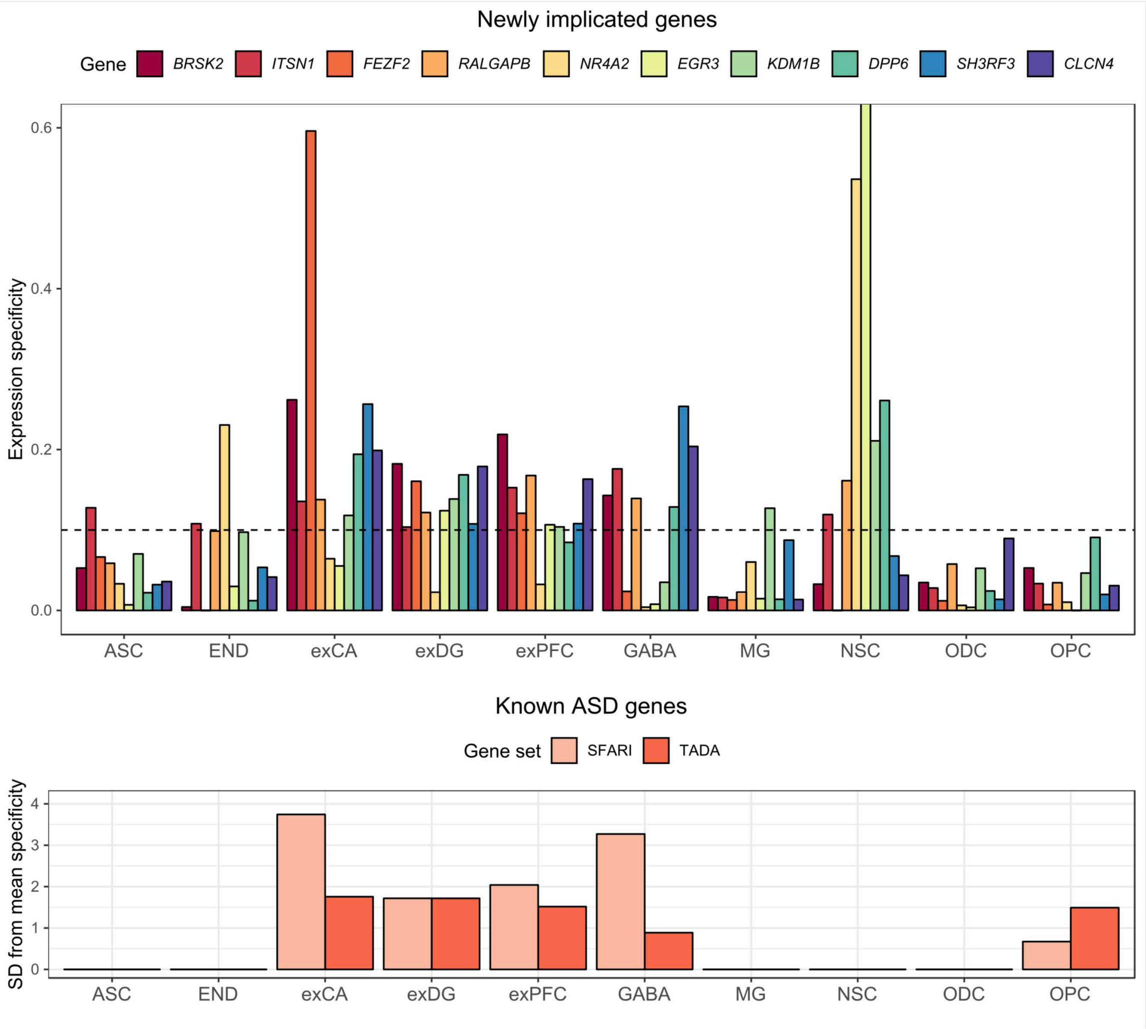

Supplementary Figure 12: Expression specificity of candidate ASD risk genes in single-cell RNA-seq data from human brains. Human neuronal cell types are defined by the single-nucleus RNA-seq data of archived human brains[50]. Known and new ASD genes were mostly enriched in neurons (exCA, exDG, exPFC) and interneurons (GABA). Highest enrichment was also observed in pyramidal neurons (exXCA). New ASD genes were also enriched in neuronal stem cells that are not implicated by known ASD genes, but the enrichment is not significant. Significance code: *= p<0.01, **= p<0.001. ASC=astrocytes, END=endothelial cells, exCA=pyramidal neurons from the hippocampus CA region, exDG=granule neurons from the Hip dentate gyrus region, exPFC=glutamatergic neurons from the PFC, GABA=GABAergic interneurons, MG=microglia, NSC=neuronal stem cells, ODC=oligodendrocytes, OPC=oligodendrocyte precursor cells.

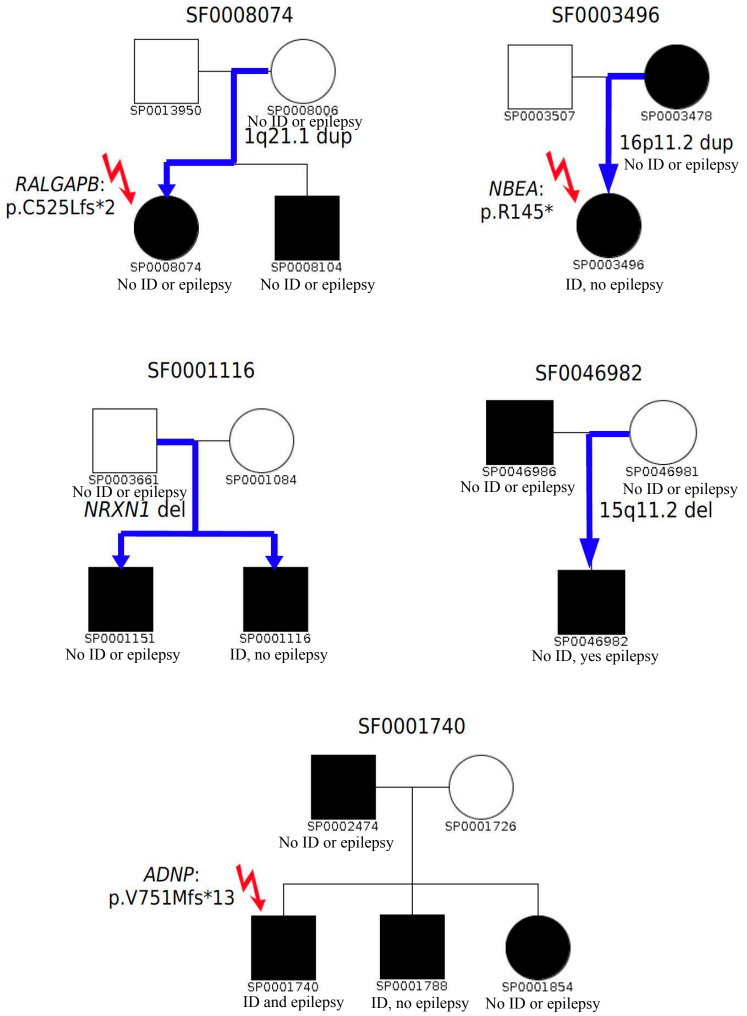

Supplementary Figure 13: Genetic causes of ASD were identified in 6 offspring in 5 multiplex families. SF0003496 has another affected offspring, who was not sequenced in this study. ID=intellectual disability.

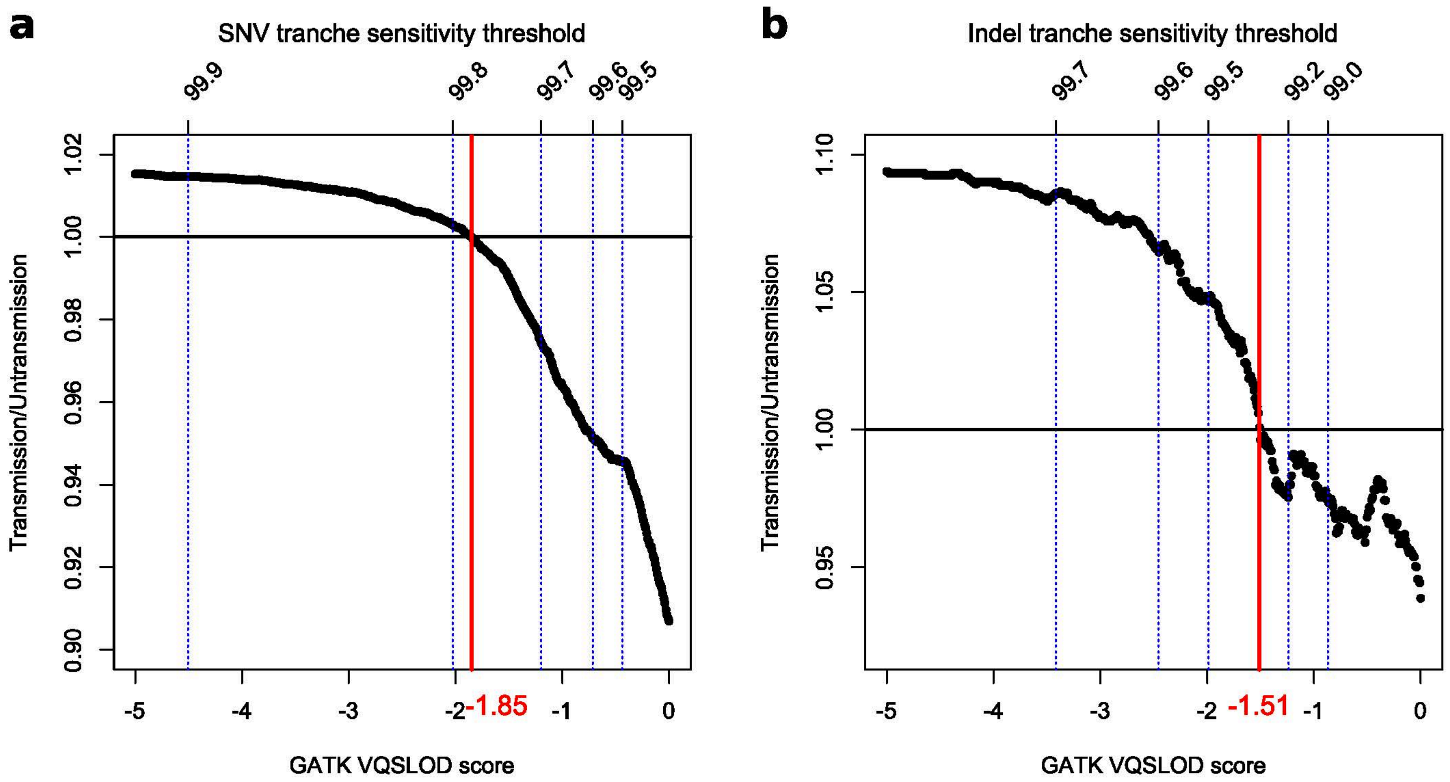

Supplementary Figure 14: Recalibrating VQS LOD threshold for analyzing inherited singleton variants. The transmission to un-transmission ratio of singleton synonymous SNVs (**a**) and non-frameshift indels (**b**) are shown as a function of the VQS LOD score. The dashed lines mark the GATK defined cutoffs based on different tranche sensitivity thresholds. The red line shows the cutoffs that balance the transmission to non-transmission ratio and were used in filtering singleton variants for transmission disequilibrium analysis.
